# Supplementary material for: The Toll‐Like Receptor Signalling Pathway Is Altered in iPSC‐Derived Cortical Networks From People With Bipolar Disorder
Source: Bipolar Disord. 2026 Aug 4;28(5):e70169. doi: 10.1111/bdi.70169 (PMC13435821; doi:10.1111/bdi.70169)
Supplement: Supplementary file 1 [file BDI-28-0-s001.pdf]

Supplementary file 4: Trilineage differentiation.

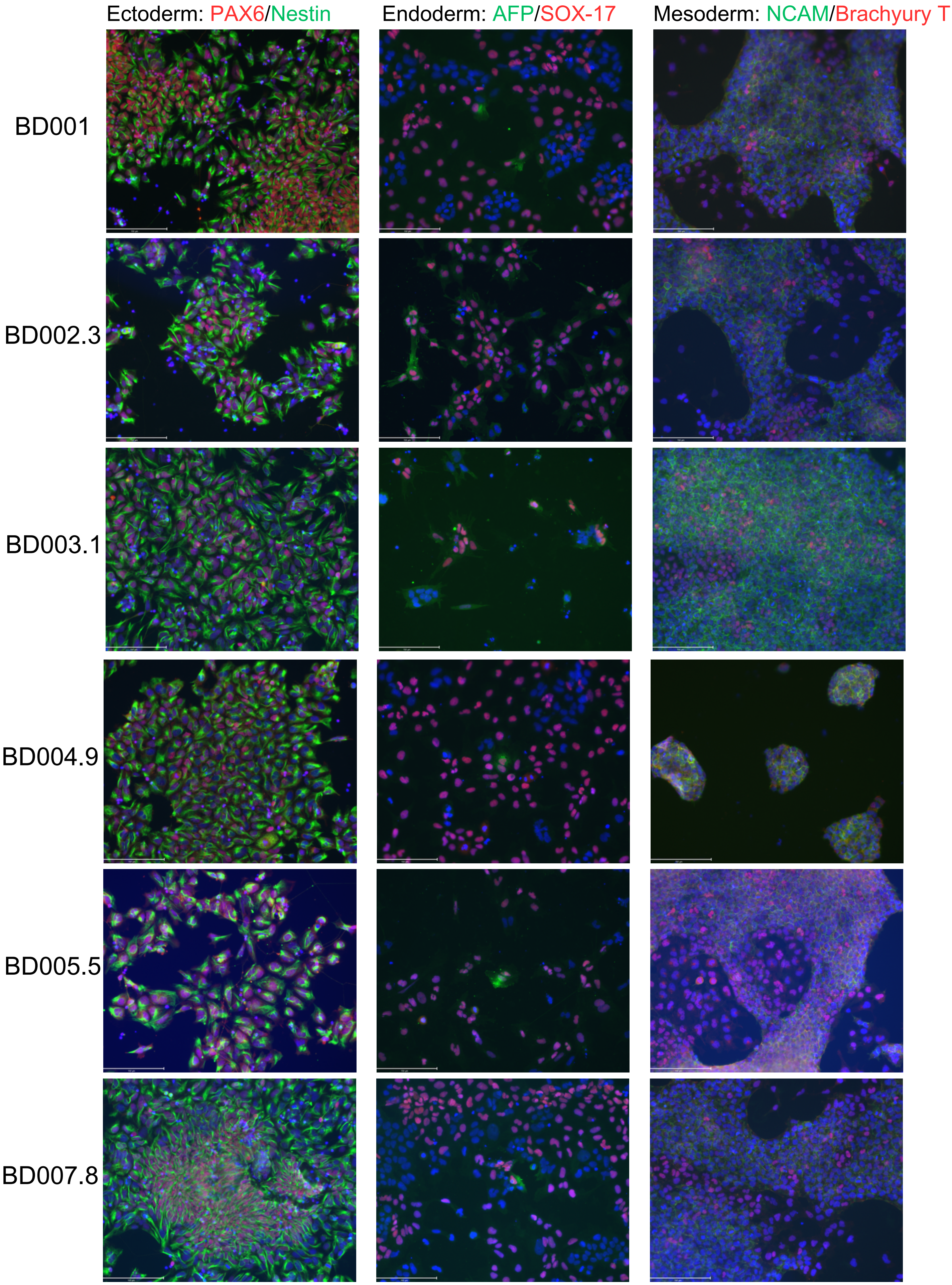

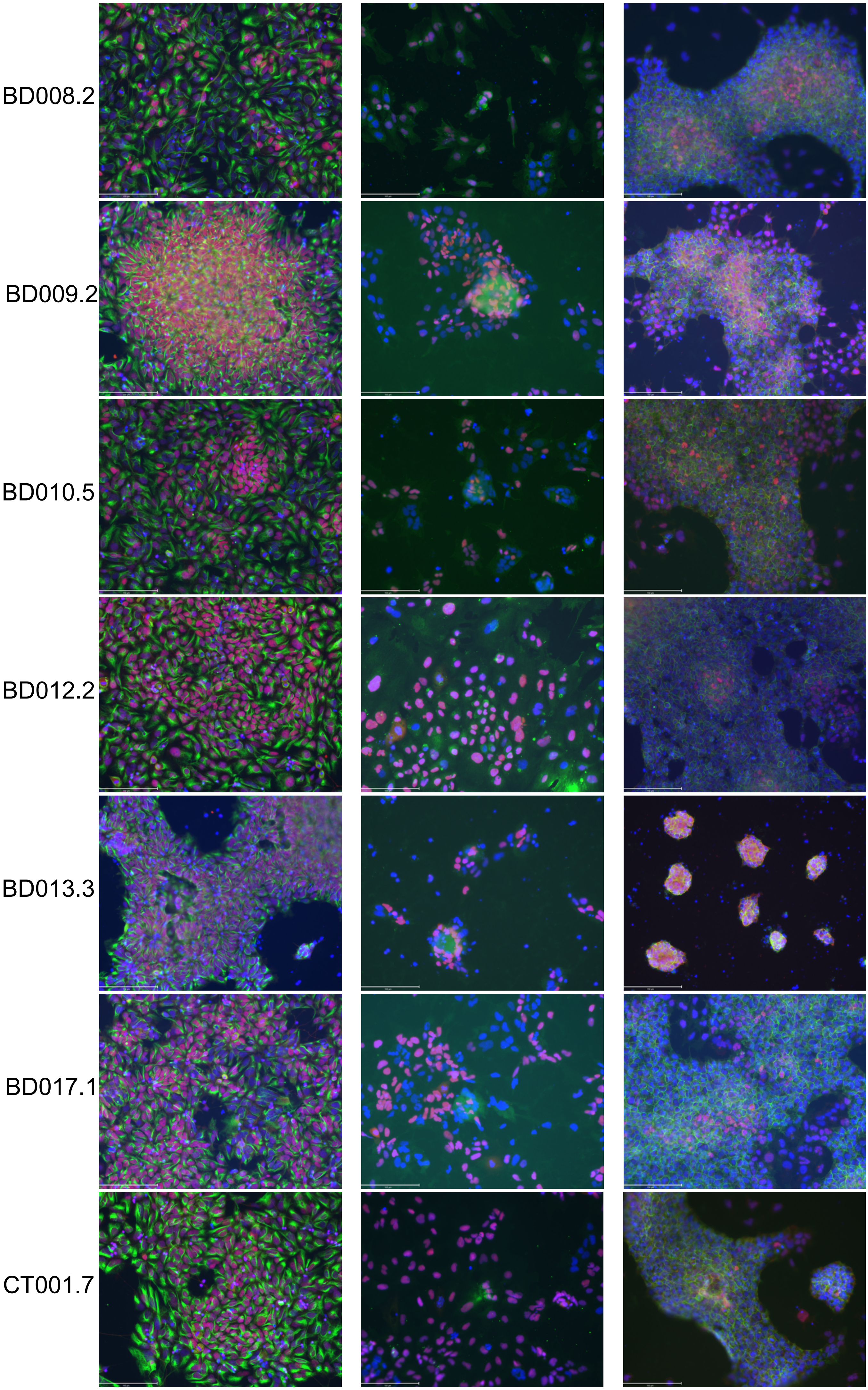
BD008.2
BD009.2
BD010.5
BD012.2
BD013.3
BD017.1
CT001.7

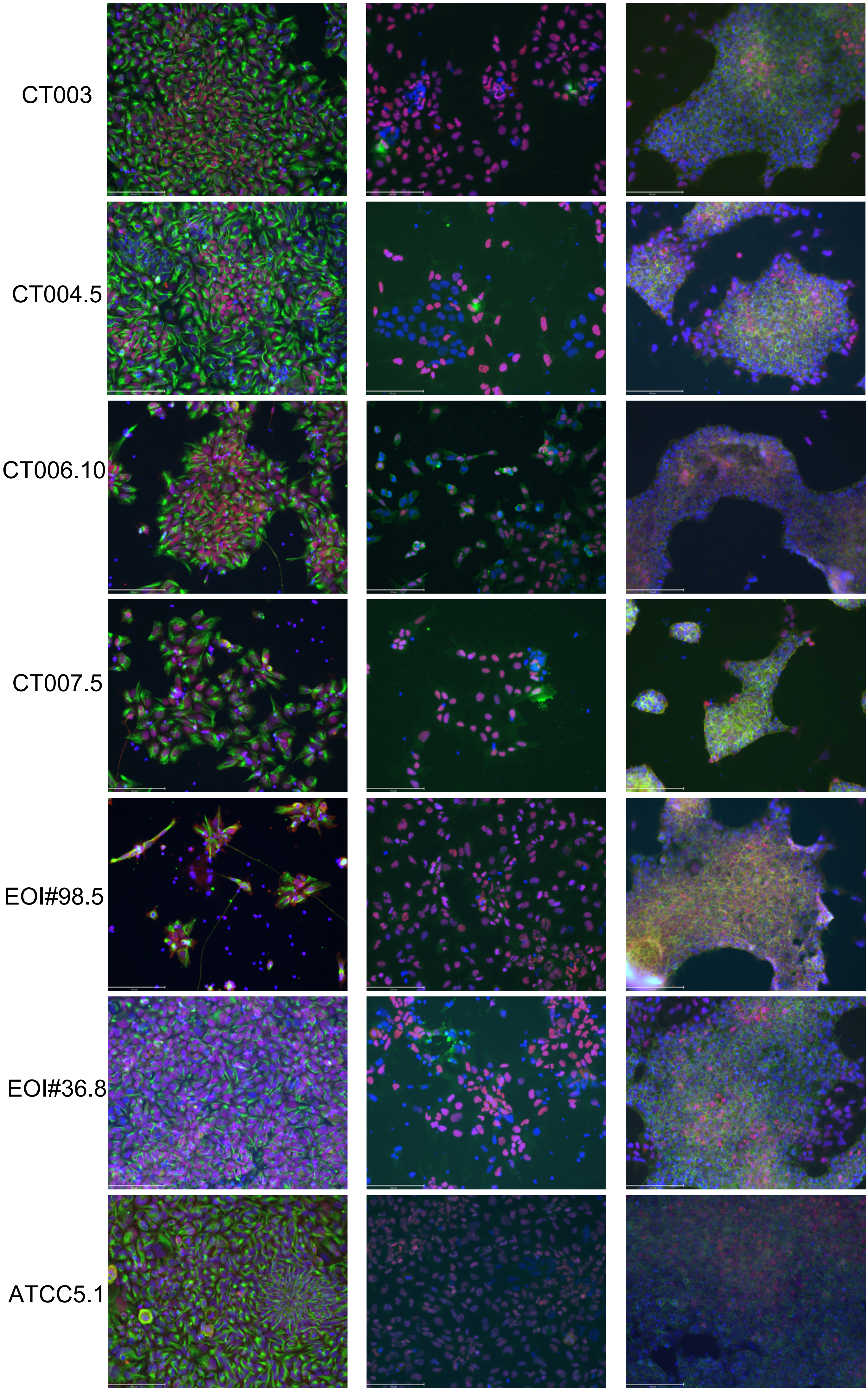

CT003
CT004.5
CT006.10
CT007.5
EOI#98.5
EOI#36.8
ATCC5.1

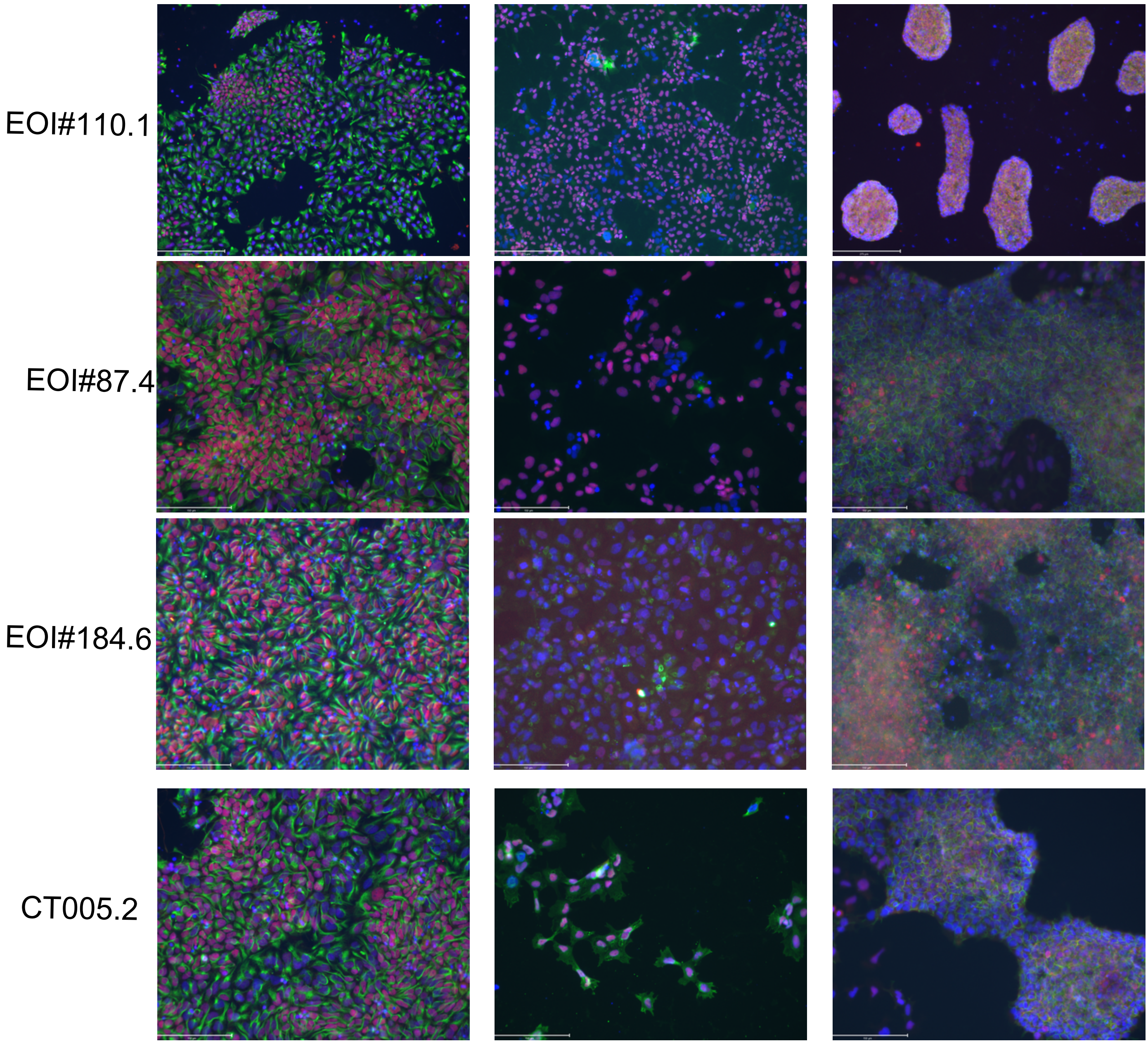

IImages of trilineage differentiation for the 24 lines generated. Immunofluorescence images for markers of the 3germ layers: Ectoderm: PAX6 - red, Nestin - green; Endoderm: AFP - green and SOX17 - red; Mesoderm: NCAM - green and BrachuryT - red. Images were taken using the EVOS M7000 microscope using a 20x objective, scale bar = 150um.
