# Supplementary material for: The Toll‐Like Receptor Signalling Pathway Is Altered in iPSC‐Derived Cortical Networks From People With Bipolar Disorder
Source: Bipolar Disord. 2026 Aug 4;28(5):e70169. doi: 10.1111/bdi.70169 (PMC13435821; doi:10.1111/bdi.70169)
Supplement: Supplementary file 2 [file BDI-28-0-s002.pdf]

Supplementary file 3: iPSCs

Phase Contrast | OCT4/SSEA4/DAPI

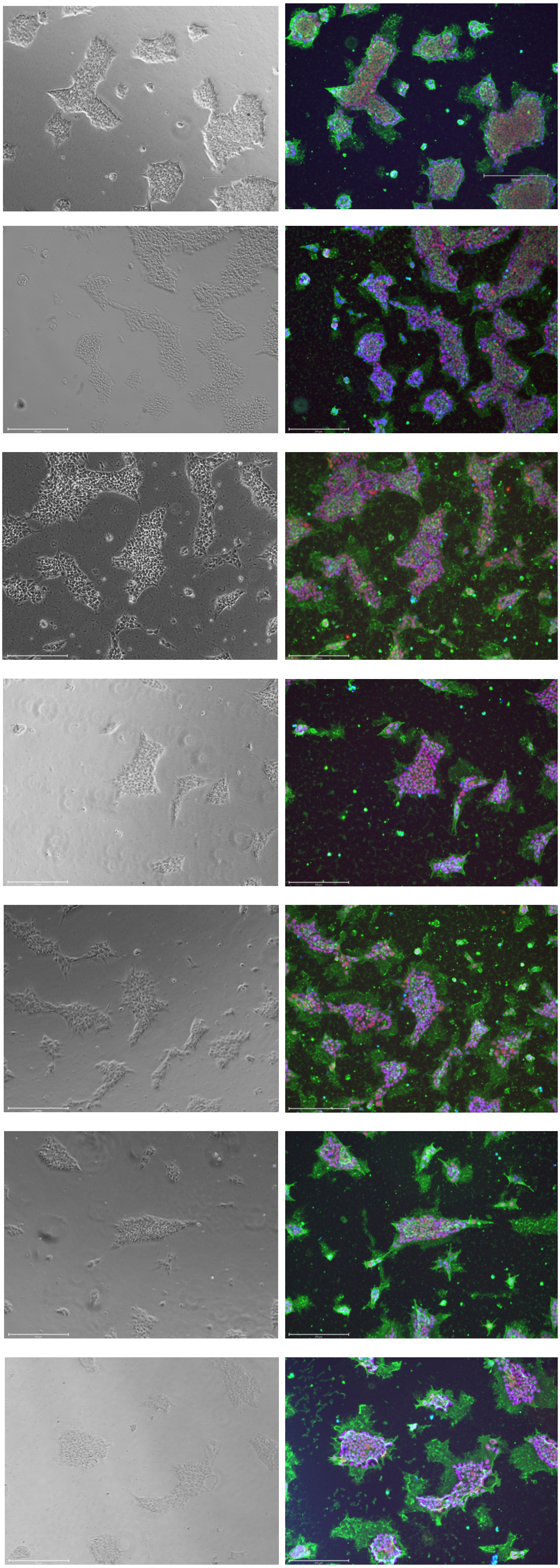

BD001

BD002.3

BD003.1

BD004.9

BD005.5

BD007.8

BD008.2

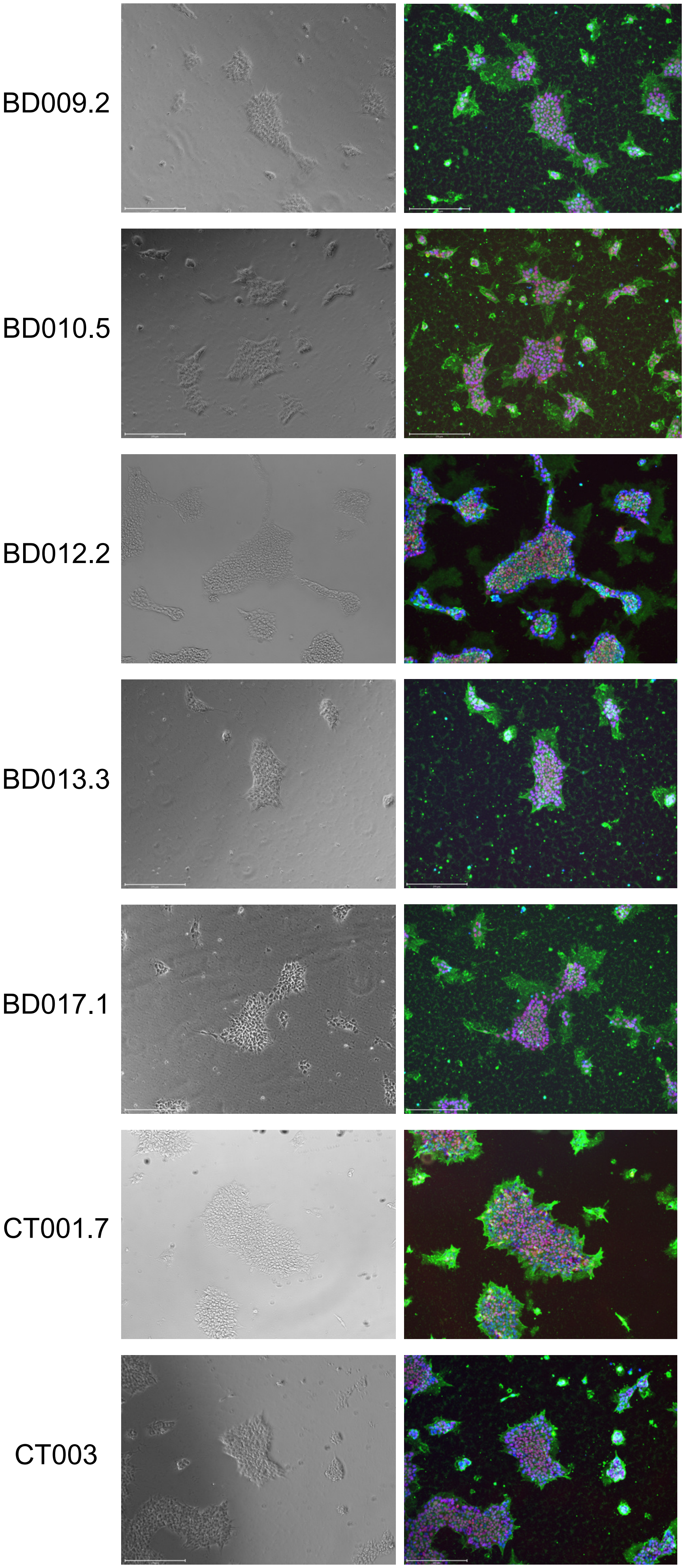
BD009.2
BD010.5
BD012.2
BD013.3
BD017.1
CT001.7
CT003

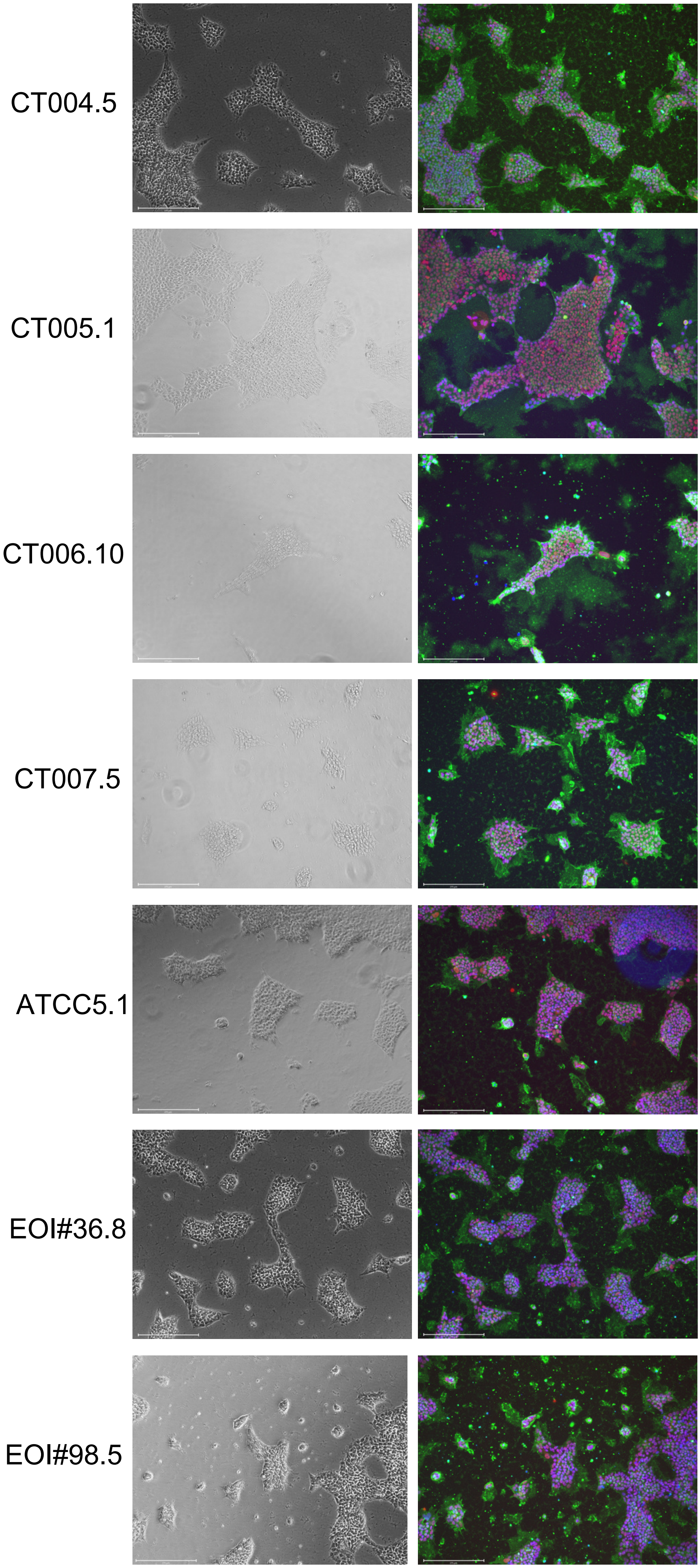
CT004.5
CT005.1
CT006.10
CT007.5
ATCC5.1
EOI#36.8
EOI#98.5

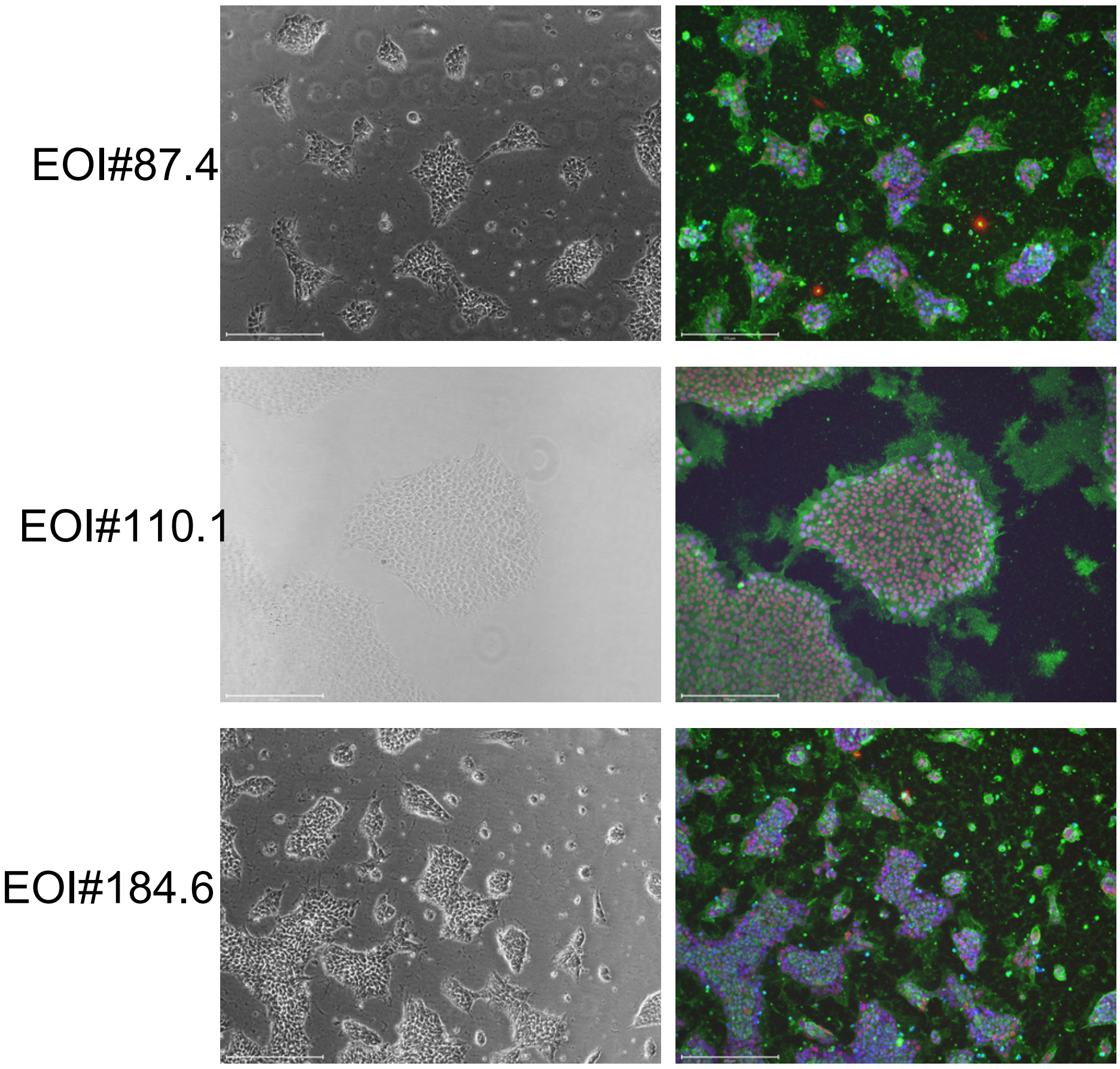

Images of the 24 iPSC lines generated. Phase contrast to observe iPSC morphology -tightly packed colonies with a high nucleus-to-cytoplasm ratio and pronounced nucleoli. Immunofluorescence image for pluripotency markers - OCT4 (red) and SSEA4 (green). Images taken with EVOS M7000 microscope using a 10x objective, scale bar = 275um.
