# Supplementary material for: The Toll‐Like Receptor Signalling Pathway Is Altered in iPSC‐Derived Cortical Networks From People With Bipolar Disorder
Source: Bipolar Disord. 2026 Aug 4;28(5):e70169. doi: 10.1111/bdi.70169 (PMC13435821; doi:10.1111/bdi.70169)
Supplement: Supplementary file 3 [file BDI-28-0-s004.pdf]

Supplementary file 2: Flow cytometry.

File contains the flow cytometry result for each individual cell line regarding the expression of iPSC markers: CD9 (FITC), SSEA4 (Pe-Cy7) and EPCAM (BV). Light grey graph represent the cells stained with isotype control antibodies, dark grey graph represent the cell stained with the primary antibodies. All lines except BD005.5 had over 90% positive for each marker.

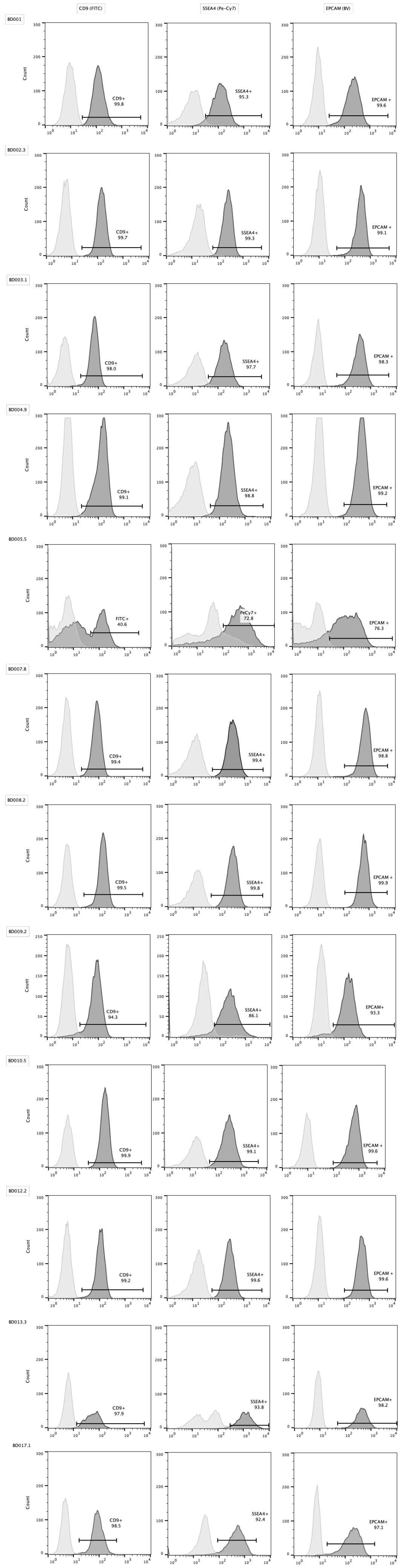
CD9 (FITC)
SSEA4 (Pe-Cy7)
EPCAM (BV)
BD001
CD9+ 99.8
SSEA4+ 95.3
EPCAM + 99.6
BD002.3
CD9+ 99.7
SSEA4+ 99.3
EPCAM + 99.1
BD003.1
CD9+ 98.0
SSEA4+ 97.7
EPCAM + 98.3
BD004.9
CD9+ 99.1
SSEA4+ 98.8
EPCAM + 99.2
BD005.5
FITC+ 40.6
PeCy7+ 72.8
EPCAM + 76.3
BD007.8
CD9+ 99.4
SSEA4+ 99.4
EPCAM + 98.8
BD008.2
CD9+ 99.5
SSEA4+ 99.8
EPCAM + 99.9
BD009.2
CD9+ 94.3
SSEA4+ 86.1
EPCAM+ 93.3
BD010.5
CD9+ 99.9
SSEA4+ 99.1
EPCAM + 99.6
BD012.2
CD9+ 99.2
SSEA4+ 99.6
EPCAM + 99.6
BD013.3
CD9+ 97.9
SSEA4+ 93.8
EPCAM+ 98.2
BD017.1
CD9+ 98.5
SSEA4+ 92.4
EPCAM+ 97.1
Count

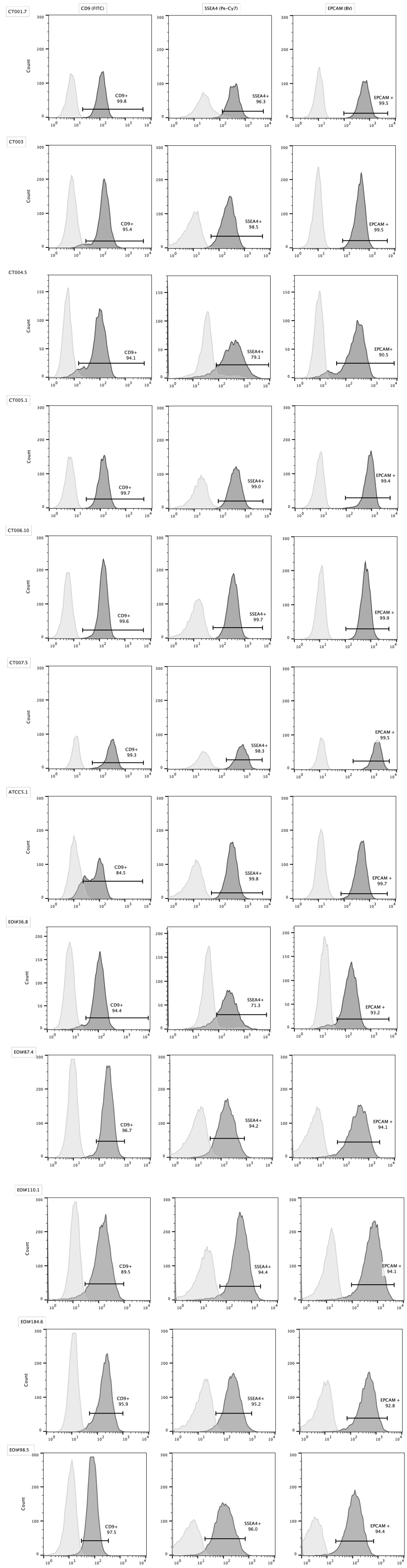
CD9 (FITC)
SSEA4 (Pe-Cy7)
EPCAM (BV)
Count
CT001.7
CD9+ 99.8
SSEA4+ 96.3
EPCAM + 99.5
CT003
CD9+ 95.4
SSEA4+ 98.5
EPCAM + 99.5
CT004.5
CD9+ 94.1
SSEA4+ 79.1
EPCAM+ 90.5
CT005.1
CD9+ 99.7
SSEA4+ 99.0
EPCAM + 99.4
CT006.10
CD9+ 99.6
SSEA4+ 99.7
EPCAM + 99.9
CT007.5
CD9+ 99.3
SSEA4+ 98.3
EPCAM + 99.5
ATCC5.1
CD9+ 84.5
SSEA4+ 99.8
EPCAM + 99.7
EOI#36.8
CD9+ 94.4
SSEA4+ 71.3
EPCAM + 93.2
EOI#87.4
CD9+ 96.7
SSEA4+ 94.2
EPCAM + 94.1
EOI#110.1
CD9+ 89.5
SSEA4+ 94.4
EPCAM + 94.1
EOI#184.6
CD9+ 95.9
SSEA4+ 95.2
EPCAM + 92.8
EOI#98.5
CD9+ 97.5
SSEA4+ 96.0
EPCAM + 94.4
