# Supplementary material for: The Toll‐Like Receptor Signalling Pathway Is Altered in iPSC‐Derived Cortical Networks From People With Bipolar Disorder
Source: Bipolar Disord. 2026 Aug 4;28(5):e70169. doi: 10.1111/bdi.70169 (PMC13435821; doi:10.1111/bdi.70169)
Supplement: Supplementary file 4 [file BDI-28-0-s005.pdf]

Supplementary file 5: Cortical networks

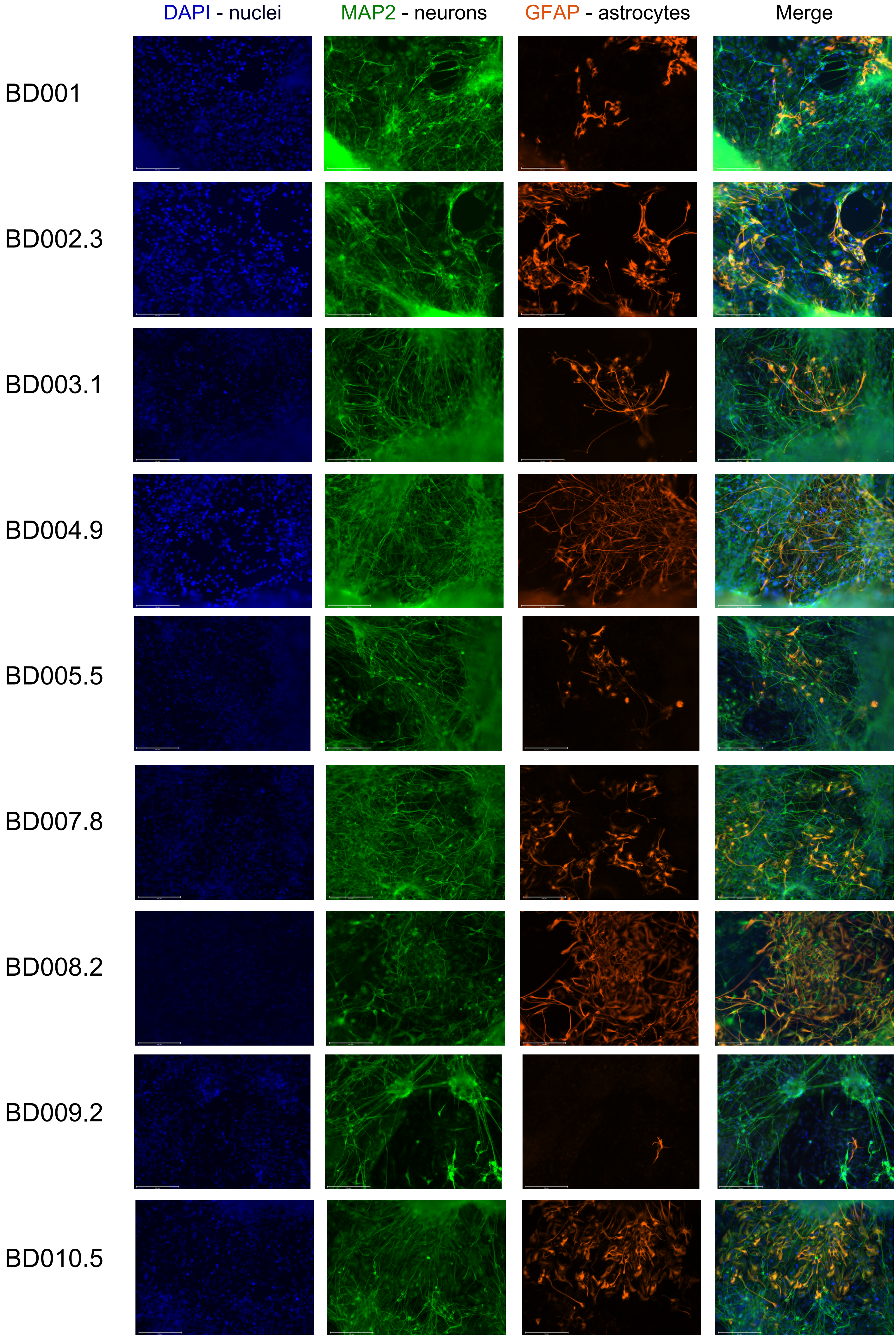

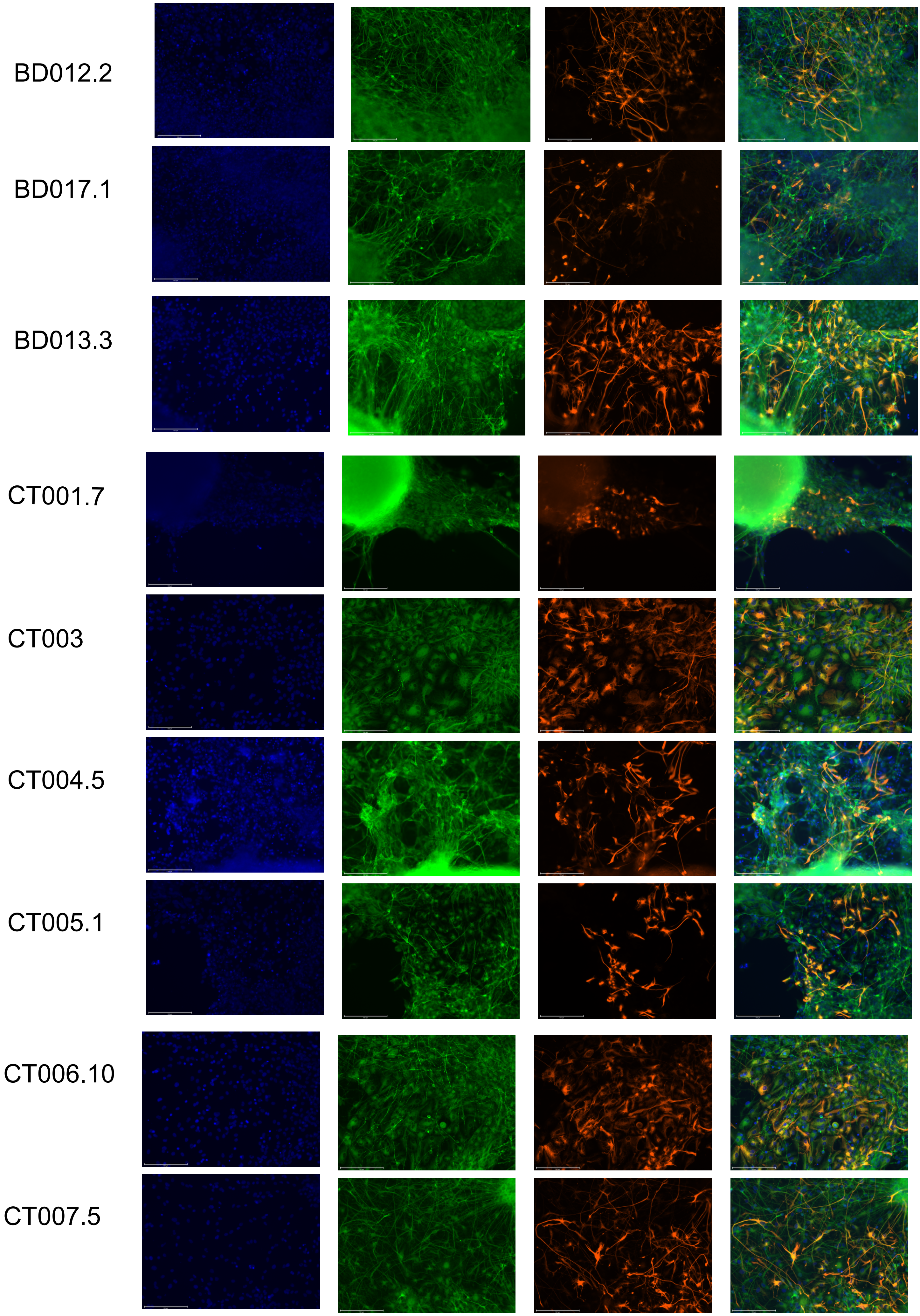
BD012.2
BD017.1
BD013.3
CT001.7
CT003
CT004.5
CT005.1
CT006.10
CT007.5

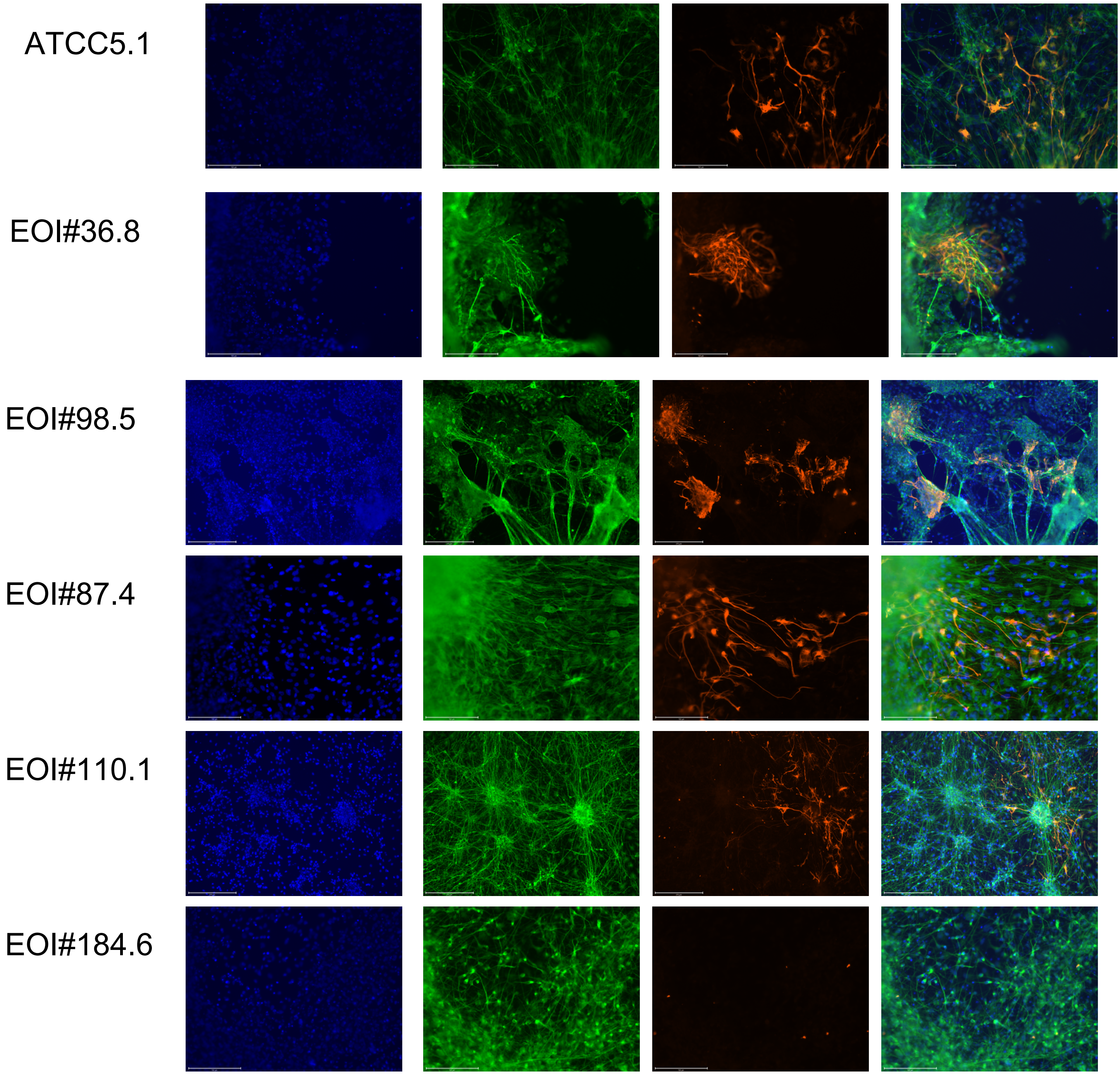


Immunofluorescence images of 24 lines differentiated into neurons (MAP2 - green) and astrocytes (GFAP - orange or purple). Images taken with EVOS M700 microscope with 20x objective, scale bar = 150um. Images are not processed, except to add a scale bar.
