# Supplementary material for: The Toll‐Like Receptor Signalling Pathway Is Altered in iPSC‐Derived Cortical Networks From People With Bipolar Disorder
Source: Bipolar Disord. 2026 Aug 4;28(5):e70169. doi: 10.1111/bdi.70169 (PMC13435821; doi:10.1111/bdi.70169)
Supplement: Supplementary file 5 [file BDI-28-0-s006.pdf]

Supplementary file 1: Karyotype reports.

This file contains the individual reports of the Illumina Infinium GSA-24 v3.0 array for each of the 24 lines reported. Lines are identified by the line ID and passage number. Report identifies the cell type submitted: cell pellet, the sex and if aneuploidies were detected. All lines have normal karyotype with no aneuploidies detected.

Victorian Clinical Genetics Services
Murdoch Children's Research Institute
Flemington Road, Parkville, VIC 3052 Australia
P +61 1300 118247 F +61 3 8341 6366
W vcgs.org.au ABN 51 007 032 760

VCGS leaders in genetic health

LIMS Report #: 901660

Patient: CT001.7 P13 Ct001.7 P13

DOB: Unknown
Sex: Unknown
VCGS sample ID: 22C103778
Date collected: 05-May-2022
Date received: 05-May-2022
Date reported: 20-Jun-2022
Source: Cell Pellet
Ext. sample ID:
Ext. patient ID:

Dr. Katerina Vlahos
Murdoch Children's Research Institute
Level 5/Flemington Road
PARKVILLE VIC 3052

CC: Ips.gene

Cytogenetics Laboratory

**Clinical details**	Cell line
**Specimen source**	Cell Pellet

Molecular karyotype

**Array type**	Illumina Infinium GSA-24 v3.0
**Resolution**	0.50Mb
**Assembly**	hg19 / GRCh37 (Feb 2009)
**Molecular karyotype**	arr(X,1-22)x2
**Result**	NO ANEUPLOIDIES DETECTED

Interpretation

Female molecular karyotype. No aneuploidies were detected in this sample.

Molecular karyotyping is limited in its ability to detect low grade mosaicism and genomic copy number changes below the resolution stated. Balanced rearrangements and Robertsonian translocations will not be detected. This test does not exclude single gene disorders caused by sequence mutations or trinucleotide repeat expansions (such as fragile X syndrome, Huntington disease, some spinocerebellar ataxias, Friedreich ataxia and myotonic dystrophy). Testing for fragile X syndrome should be considered in individuals with developmental delay/ intellectual disability. Further genomic based testing may be considered if there remains a high suspicion of a monogenic disorder. Copy number variants that do not contain genes, are well established polymorphisms, or are assessed as being of unlikely clinical significance (based on "ACMG Technical Standards for the Interpretation and Reporting of Constitutional Copy Number Variants"), will not be reported. The classification is based on the current scientific evidence available at the time of reporting. Reporting of regions of homozygosity (>5Mb) is dependent on referral setting and clinical indication. CNVs that contain autosomal recessive genes will not be reported unless there is specific clinical relevance, high carrier population frequency or a history of consanguinity. Please contact the laboratory if there is a family history of a known recessive disorder or a clinically suspected recessive condition. This testing was performed on a standard SNP microarray platform which may not have sufficient probe coverage to detect clinically relevant CNVs related to this patient's specific clinical features. Please contact the laboratory if a higher resolution microarray may be required for a specific gene or genetic condition. This test does not exclude the possibility of tissue limited mosaicism and further testing of an alternative tissue may be considered if clinically indicated. Interpretation is based on the UCSC GRCh37/hg19 human reference sequence.

Validated: 20-Jun-2022 by David Francis

Enquiries: +61 1300 11 8247

PATHOLOGY REPORT

END OF TEST REPORT

FINAL REPORT

Victorian Clinical Genetics Services
Murdoch Children's Research Institute
Flemington Road, Parkville, VIC 3052 Australia
P +61 1300 118247 F +61 3 8341 6366
W vcgs.org.au ABN 51 007 032 760

leaders in genetic health

LIMS Report #: 901655

Patient: CT004.5 P12 Ct004.5 P12

Dr. Katerina Vlahos
Murdoch Children's Research Institute
Level 5/Flemington Road
PARKVILLE VIC 3052

DOB: Unknown
Sex: Unknown
VCGS sample ID: 22C103776
Date collected: 05-May-2022
Date received: 05-May-2022
Date reported: 20-Jun-2022
Source: Cell Pellet
Ext. sample ID:
Ext. patient ID:

CC: Ips.gene

Cytogenetics Laboratory

**Clinical details**	Cell line
**Specimen source**	Cell Pellet

Molecular karyotype

**Array type**	Illumina Infinium GSA-24 v3.0
**Resolution**	0.50Mb
**Assembly**	hg19 / GRCh37 (Feb 2009)
**Molecular karyotype**	arr(X,1-22)x2
**Result**	NO ANEUPLOIDIES DETECTED

Interpretation

Female molecular karyotype. No aneuploidies were detected in this sample.

Molecular karyotyping is limited in its ability to detect low grade mosaicism and genomic copy number changes below the resolution stated. Balanced rearrangements and Robertsonian translocations will not be detected. This test does not exclude single gene disorders caused by sequence mutations or trinucleotide repeat expansions (such as fragile X syndrome, Huntington disease, some spinocerebellar ataxias, Friedreich ataxia and myotonic dystrophy). Testing for fragile X syndrome should be considered in individuals with developmental delay/ intellectual disability. Further genomic based testing may be considered if there remains a high suspicion of a monogenic disorder. Copy number variants that do not contain genes, are well established polymorphisms, or are assessed as being of unlikely clinical significance (based on "ACMG Technical Standards for the Interpretation and Reporting of Constitutional Copy Number Variants"), will not be reported. The classification is based on the current scientific evidence available at the time of reporting. Reporting of regions of homozygosity (>5Mb) is dependent on referral setting and clinical indication. CNVs that contain autosomal recessive genes will not be reported unless there is specific clinical relevance, high carrier population frequency or a history of consanguinity. Please contact the laboratory if there is a family history of a known recessive disorder or a clinically suspected recessive condition. This testing was performed on a standard SNP microarray platform which may not have sufficient probe coverage to detect clinically relevant CNVs related to this patient's specific clinical features. Please contact the laboratory if a higher resolution microarray may be required for a specific gene or genetic condition. This test does not exclude the possibility of tissue limited mosaicism and further testing of an alternative tissue may be considered if clinically indicated. Interpretation is based on the UCSC GRCh37/hg19 human reference sequence.

Validated: 20-Jun-2022 by David Francis

Enquiries: +61 1300 11 8247

END OF TEST REPORT

FINAL REPORT

PATHOLOGY REPORT

The Royal College of Pathologists of Australasia
Accredited for compliance with NPAAC Standards and ISO 15189
Accreditation number: 3171

Victorian Clinical Genetics Services
Murdoch Children's Research Institute
Flemington Road, Parkville, VIC 3052 Australia
P +61 1300 118247 F +61 3 8341 6366
W vcgs.org.au ABN 51 007 032 760

LIMS Report #: 850334

Patient: CT005.1 P10 Ct005.1 P10

DOB: Unknown
Sex: Unknown
VCGS sample ID: 21C111364
Date collected: 14-Dec-2021
Date received: 14-Dec-2021
Date reported: 05-Jan-2022
Source: Cell Pellet
Ext. sample ID:
Ext. patient ID:

Dr. Katerina Vlahos
Murdoch Childrens Research Institute
Level 5/Flemington Road
PARKVILLE VIC 3052

Cytogenetics Laboratory

**Clinical details**	Cell line
**Specimen source**	Cell Pellet

Molecular karyotype

**Array type**	Illumina Infinium GSA-24 v3.0
**Resolution**	0.50Mb
**Assembly**	hg19 / GRCh37 (Feb 2009)
**Molecular karyotype**	arr(X,1-22)x2
**Result**	NO ANEUPLOIDIES DETECTED

Interpretation

Female molecular karyotype. No aneuploidies were detected in this sample.

Molecular karyotyping is limited in its ability to detect low grade mosaicism and genomic copy number changes below the resolution stated. Balanced rearrangements and Robertsonian translocations will not be detected. This test does not exclude single gene disorders caused by sequence mutations or trinucleotide repeat expansions (such as fragile X syndrome, Huntington disease, some spinocerebellar ataxias, Friedreich ataxia and myotonic dystrophy). Testing for fragile X syndrome should be considered in individuals with developmental delay/ intellectual disability. Further genomic based testing may be considered if there remains a high suspicion of a monogenic disorder. Copy number variants that do not contain genes, are well established polymorphisms, or are assessed as being of unlikely clinical significance (based on "ACMG Technical Standards for the Interpretation and Reporting of Constitutional Copy Number Variants"), will not be reported. The classification is based on the current scientific evidence available at the time of reporting. Reporting of regions of homozygosity (>5Mb) is dependent on referral setting and clinical indication. CNVs that contain autosomal recessive genes will not be reported unless there is specific clinical relevance, high carrier population frequency or a history of consanguinity. Please contact the laboratory if there is a family history of a known recessive disorder or a clinically suspected recessive condition. This testing was performed on a standard SNP microarray platform which may not have sufficient probe coverage to detect clinically relevant CNVs related to this patient's specific clinical features. Please contact the laboratory if a higher resolution microarray may be required for a specific gene or genetic condition. This test does not exclude the possibility of tissue limited mosaicism and further testing of an alternative tissue may be considered if clinically indicated. Interpretation is based on the UCSC GRCh37/hg19 human reference sequence.

Validated: 05-Jan-2022 by David Francis

Enquiries: +61 1300 11 8247

PATHOLOGY REPORT

END OF TEST REPORT

FINAL REPORT

Victorian Clinical Genetics Services
Murdoch Children's Research Institute
Flemington Road, Parkville, VIC 3052 Australia
P +61 1300 118247 F +61 3 8341 6366
W vcgs.org.au ABN 51 007 032 760

LIMS Report #: 850335

Patient: CT006.10 P10 Ct006.10 P10

Dr. Katerina Vlahos
Murdoch Childrens Research Institute
Level 5/Flemington Road
PARKVILLE VIC 3052

DOB: Unknown
Sex: Unknown
VCGS sample ID: 21C111365
Date collected: 14-Dec-2021
Date received: 14-Dec-2021
Date reported: 05-Jan-2022
Source: Cell Pellet
Ext. sample ID:
Ext. patient ID:

Cytogenetics Laboratory

PATHOLOGY REPORT

**Clinical details**	Cell line
**Specimen source**	Cell Pellet

Molecular karyotype

**Array type**	Illumina Infinium GSA-24 v3.0
**Resolution**	0.50Mb
**Assembly**	hg19 / GRCh37 (Feb 2009)
**Molecular karyotype**	arr(X,1-22)x2
**Result**	NO ANEUPLOIDIES DETECTED

Interpretation

Female molecular karyotype. No aneuploidies were detected in this sample.

Molecular karyotyping is limited in its ability to detect low grade mosaicism and genomic copy number changes below the resolution stated. Balanced rearrangements and Robertsonian translocations will not be detected. This test does not exclude single gene disorders caused by sequence mutations or trinucleotide repeat expansions (such as fragile X syndrome, Huntington disease, some spinocerebellar ataxias, Friedreich ataxia and myotonic dystrophy). Testing for fragile X syndrome should be considered in individuals with developmental delay/ intellectual disability. Further genomic based testing may be considered if there remains a high suspicion of a monogenic disorder. Copy number variants that do not contain genes, are well established polymorphisms, or are assessed as being of unlikely clinical significance (based on "ACMG Technical Standards for the Interpretation and Reporting of Constitutional Copy Number Variants"), will not be reported. The classification is based on the current scientific evidence available at the time of reporting. Reporting of regions of homozygosity (>5Mb) is dependent on referral setting and clinical indication. CNVs that contain autosomal recessive genes will not be reported unless there is specific clinical relevance, high carrier population frequency or a history of consanguinity. Please contact the laboratory if there is a family history of a known recessive disorder or a clinically suspected recessive condition. This testing was performed on a standard SNP microarray platform which may not have sufficient probe coverage to detect clinically relevant CNVs related to this patient's specific clinical features. Please contact the laboratory if a higher resolution microarray may be required for a specific gene or genetic condition. This test does not exclude the possibility of tissue limited mosaicism and further testing of an alternative tissue may be considered if clinically indicated. Interpretation is based on the UCSC GRCh37/hg19 human reference sequence.

Validated: 05-Jan-2022 by David Francis

Enquiries: +61 1300 11 8247

END OF TEST REPORT

FINAL REPORT

Accredited for compliance with NPAAC Standards and ISO 15189
Accreditation number: 3171

Victorian Clinical Genetics Services
Murdoch Children's Research Institute
Flemington Road, Parkville, VIC 3052 Australia
P +61 1300 118247 F +61 3 8341 6366
W vcgs.org.au ABN 51 007 032 760

leaders in genetic health

LIMS Report #: 1011170

Patient: #36.8 IPSC P11 #36.8 Ipsc P11

Dr. Katerina Vlahos
Murdoch Children's Research Institute
Level 5/Flemington Road
PARKVILLE VIC 3052

DOB: Unknown
Sex:
VCGS sample ID: 23C105302
Date collected: 10-May-2023
Date received: 10-May-2023
Date reported: 06-Jun-2023
Source: Cell Pellet
Ext. sample ID:
Ext. patient ID:

Cytogenetics Laboratory

**Clinical details**	Cell line
**Specimen source**	Cell Pellet

Molecular karyotype

**Array type**	Illumina Infinium GSA-24 v3.0
**Resolution**	0.50Mb
**Reference genome**	GRCh38 / hg38 (Dec 2013)
**Molecular karyotype**	arr(X,Y)x1,(1-22)x2
**Result**	NO ANEUPLOIDIES DETECTED

Interpretation

Male molecular karyotype. No aneuploidies were detected in this sample.

Molecular karyotyping is limited in its ability to detect low grade mosaicism and genomic copy number changes below the resolution stated. Balanced rearrangements and Robertsonian translocations will not be detected. This test does not exclude single gene disorders caused by sequence mutations or trinucleotide repeat expansions (such as fragile X syndrome, Huntington disease, some spinocerebellar ataxias, Friedreich ataxia and myotonic dystrophy). Testing for fragile X syndrome should be considered in individuals with developmental delay/ intellectual disability. Further genomic based testing may be considered if there remains a high suspicion of a monogenic disorder. Copy number variants that do not contain genes, are well established polymorphisms, or are assessed as being of unlikely clinical significance (based on "ACMG Technical Standards for the Interpretation and Reporting of Constitutional Copy Number Variants"), will not be reported. The classification is based on the current scientific evidence available at the time of reporting. Reporting of regions of homozygosity (>5Mb) is dependent on referral setting and clinical indication. CNVs that contain autosomal recessive genes will not be reported unless there is specific clinical relevance, high carrier population frequency or a history of consanguinity. Please contact the laboratory if there is a family history of a known recessive disorder or a clinically suspected recessive condition. CNVs involving moderate to high risk cancer susceptibility genes may be reported. However, incidentally ascertained CNVs involving genes assessed as having low to moderate cancer risk will not generally be reported unless they are part of a larger CNV. This testing was performed on a standard SNP microarray platform which may not have sufficient probe coverage to detect clinically relevant CNVs related to this patient's specific clinical features. Please contact the laboratory if a higher resolution microarray may be required for a specific gene or genetic condition. This test does not exclude the possibility of tissue limited mosaicism and further testing of an alternative tissue may be considered if clinically indicated. Interpretation is based on the UCSC GRCh38/hg38 human reference sequence.

Validated: 06-Jun-2023 by David Francis

Enquiries: +61 1300 11 8247

PATHOLOGY REPORT

END OF TEST REPORT

FINAL REPORT

Victorian Clinical Genetics Services
Murdoch Children's Research Institute
Flemington Road, Parkville, VIC 3052 Australia
P +61 1300 118247 F +61 3 8341 6366
W vcgs.org.au ABN 51 007 032 760

VCGS leaders in genetic health

LIMS Report #: 850331

Patient: CT003 P12 Ct003 P12

Dr. Katerina Vlahos
Murdoch Childrens Research Institute
Level 5/Flemington Road
PARKVILLE VIC 3052

DOB: Unknown
Sex: Unknown
VCGS sample ID: 21C111362
Date collected: 14-Dec-2021
Date received: 14-Dec-2021
Date reported: 05-Jan-2022
Source: Cell Pellet
Ext. sample ID:
Ext. patient ID:

Cytogenetics Laboratory

**Clinical details**	Cell line
**Specimen source**	Cell Pellet

Molecular karyotype

**Array type**	Illumina Infinium GSA-24 v3.0
**Resolution**	0.50Mb
**Assembly**	hg19 / GRCh37 (Feb 2009)
**Molecular karyotype**	arr(X,1-22)x2
**Result**	NO ANEUPLOIDIES DETECTED

Interpretation

Female molecular karyotype. No aneuploidies were detected in this sample.

Molecular karyotyping is limited in its ability to detect low grade mosaicism and genomic copy number changes below the resolution stated. Balanced rearrangements and Robertsonian translocations will not be detected. This test does not exclude single gene disorders caused by sequence mutations or trinucleotide repeat expansions (such as fragile X syndrome, Huntington disease, some spinocerebellar ataxias, Friedreich ataxia and myotonic dystrophy). Testing for fragile X syndrome should be considered in individuals with developmental delay/ intellectual disability. Further genomic based testing may be considered if there remains a high suspicion of a monogenic disorder. Copy number variants that do not contain genes, are well established polymorphisms, or are assessed as being of unlikely clinical significance (based on "ACMG Technical Standards for the Interpretation and Reporting of Constitutional Copy Number Variants"), will not be reported. The classification is based on the current scientific evidence available at the time of reporting. Reporting of regions of homozygosity (>5Mb) is dependent on referral setting and clinical indication. CNVs that contain autosomal recessive genes will not be reported unless there is specific clinical relevance, high carrier population frequency or a history of consanguinity. Please contact the laboratory if there is a family history of a known recessive disorder or a clinically suspected recessive condition. This testing was performed on a standard SNP microarray platform which may not have sufficient probe coverage to detect clinically relevant CNVs related to this patient's specific clinical features. Please contact the laboratory if a higher resolution microarray may be required for a specific gene or genetic condition. This test does not exclude the possibility of tissue limited mosaicism and further testing of an alternative tissue may be considered if clinically indicated. Interpretation is based on the UCSC GRCh37/hg19 human reference sequence.

Validated: 05-Jan-2022 by David Francis

Enquiries: +61 1300 11 8247

PATHOLOGY REPORT

END OF TEST REPORT

FINAL REPORT

Victorian Clinical Genetics Services
Murdoch Children's Research Institute
Flemington Road, Parkville, VIC 3052 Australia
P +61 1300 118247 F +61 3 8341 6366
W vcgs.org.au ABN 51 007 032 760

LIMS Report #: 850336

Patient: CT007.5 P10 Ct007.5 P10

Dr. Katerina Vlahos
Murdoch Childrens Research Institute
Level 5/Flemington Road
PARKVILLE VIC 3052

DOB: Unknown
Sex: Unknown
VCGS sample ID: 21C111366
Date collected: 14-Dec-2021
Date received: 14-Dec-2021
Date reported: 05-Jan-2022
Source: Cell Pellet
Ext. sample ID:
Ext. patient ID:

Cytogenetics Laboratory

**Clinical details**	Cell line
**Specimen source**	Cell Pellet

Molecular karyotype

**Array type**	Illumina Infinium GSA-24 v3.0
**Resolution**	0.50Mb
**Assembly**	hg19 / GRCh37 (Feb 2009)
**Molecular karyotype**	arr(X,1-22)x2
**Result**	NO ANEUPLOIDIES DETECTED

Interpretation

Female molecular karyotype. No aneuploidies were detected in this sample.

Molecular karyotyping is limited in its ability to detect low grade mosaicism and genomic copy number changes below the resolution stated. Balanced rearrangements and Robertsonian translocations will not be detected. This test does not exclude single gene disorders caused by sequence mutations or trinucleotide repeat expansions (such as fragile X syndrome, Huntington disease, some spinocerebellar ataxias, Friedreich ataxia and myotonic dystrophy). Testing for fragile X syndrome should be considered in individuals with developmental delay/ intellectual disability. Further genomic based testing may be considered if there remains a high suspicion of a monogenic disorder. Copy number variants that do not contain genes, are well established polymorphisms, or are assessed as being of unlikely clinical significance (based on "ACMG Technical Standards for the Interpretation and Reporting of Constitutional Copy Number Variants"), will not be reported. The classification is based on the current scientific evidence available at the time of reporting. Reporting of regions of homozygosity (>5Mb) is dependent on referral setting and clinical indication. CNVs that contain autosomal recessive genes will not be reported unless there is specific clinical relevance, high carrier population frequency or a history of consanguinity. Please contact the laboratory if there is a family history of a known recessive disorder or a clinically suspected recessive condition. This testing was performed on a standard SNP microarray platform which may not have sufficient probe coverage to detect clinically relevant CNVs related to this patient's specific clinical features. Please contact the laboratory if a higher resolution microarray may be required for a specific gene or genetic condition. This test does not exclude the possibility of tissue limited mosaicism and further testing of an alternative tissue may be considered if clinically indicated. Interpretation is based on the UCSC GRCh37/hg19 human reference sequence.

Validated: 05-Jan-2022 by David Francis

Enquiries: +61 1300 11 8247

PATHOLOGY REPORT

END OF TEST REPORT

FINAL REPORT

Victorian Clinical Genetics Services
Murdoch Children's Research Institute
Flemington Road, Parkville, VIC 3052 Australia
P +61 1300 118247 F +61 3 8341 6366
W vcgs.org.au ABN 51 007 032 760

leaders in genetic health

LIMS Report #: 850329

Patient: ATCC 5.1 P10 Atcc 5.1 P10

Dr. Katerina Vlahos
Murdoch Childrens Research Institute
Level 5/Flemington Road
PARKVILLE VIC 3052

DOB: Unknown
Sex: Unknown
VCGS sample ID: 21C111360
Date collected: 14-Dec-2021
Date received: 14-Dec-2021
Date reported: 05-Jan-2022
Source: Cell Pellet
Ext. sample ID:
Ext. patient ID:

Cytogenetics Laboratory

**Clinical details**	Cell line
**Specimen source**	Cell Pellet

Molecular karyotype

**Array type**	Illumina Infinium GSA-24 v3.0
**Resolution**	0.50Mb
**Assembly**	hg19 / GRCh37 (Feb 2009)
**Molecular karyotype**	arr(X,1-22)x2
**Result**	NO ANEUPLOIDIES DETECTED

Interpretation

Female molecular karyotype. No aneuploidies were detected in this sample.

Molecular karyotyping is limited in its ability to detect low grade mosaicism and genomic copy number changes below the resolution stated. Balanced rearrangements and Robertsonian translocations will not be detected. This test does not exclude single gene disorders caused by sequence mutations or trinucleotide repeat expansions (such as fragile X syndrome, Huntington disease, some spinocerebellar ataxias, Friedreich ataxia and myotonic dystrophy). Testing for fragile X syndrome should be considered in individuals with developmental delay/ intellectual disability. Further genomic based testing may be considered if there remains a high suspicion of a monogenic disorder. Copy number variants that do not contain genes, are well established polymorphisms, or are assessed as being of unlikely clinical significance (based on "ACMG Technical Standards for the Interpretation and Reporting of Constitutional Copy Number Variants"), will not be reported. The classification is based on the current scientific evidence available at the time of reporting. Reporting of regions of homozygosity (>5Mb) is dependent on referral setting and clinical indication. CNVs that contain autosomal recessive genes will not be reported unless there is specific clinical relevance, high carrier population frequency or a history of consanguinity. Please contact the laboratory if there is a family history of a known recessive disorder or a clinically suspected recessive condition. This testing was performed on a standard SNP microarray platform which may not have sufficient probe coverage to detect clinically relevant CNVs related to this patient's specific clinical features. Please contact the laboratory if a higher resolution microarray may be required for a specific gene or genetic condition. This test does not exclude the possibility of tissue limited mosaicism and further testing of an alternative tissue may be considered if clinically indicated. Interpretation is based on the UCSC GRCh37/hg19 human reference sequence.

Validated: 05-Jan-2022 by David Francis

Enquiries: +61 1300 11 8247

PATHOLOGY REPORT

END OF TEST REPORT

FINAL REPORT

Victorian Clinical Genetics Services
Murdoch Children's Research Institute
Flemington Road, Parkville, VIC 3052 Australia
P +61 1300 118247 F +61 3 8341 6366
W vcgs.org.au ABN 51 007 032 760

LIMS Report #: 1061935

Patient: EOI #184.6 P11 Eoi #184.6 P11

DOB: Unknown
Sex: Unknown
VCGS sample ID: 23C112728
Date collected: 19-Oct-2023
Date received: 19-Oct-2023
Date reported: 10-Nov-2023
Source: Cell Pellet
Ext. sample ID:
Ext. patient ID:

Dr. Katerina Vlahos
Murdoch Children's Research Institute
Level 5/Flemington Road
PARKVILLE VIC 3052

CC: Ips.gene

Cytogenetics Laboratory

**Clinical details**	Cell line
**Specimen source**	Cell Pellet

Molecular karyotype

**Array type**	Illumina Infinium GSA-24 v3.0
**Resolution**	0.50Mb
**Reference genome**	GRCh38 / hg38 (Dec 2013)
**Molecular karyotype**	arr(X,1-22)x2
**Result**	NO ANEUPLOIDIES DETECTED

Interpretation

Female molecular karyotype. No aneuploidies were detected in this sample.

Molecular karyotyping is limited in its ability to detect low grade mosaicism and genomic copy number changes below the resolution stated. Balanced rearrangements and Robertsonian translocations will not be detected. This test does not exclude single gene disorders caused by sequence mutations or trinucleotide repeat expansions (such as fragile X syndrome, Huntington disease, some spinocerebellar ataxias, Friedreich ataxia and myotonic dystrophy). Testing for fragile X syndrome should be considered in individuals with developmental delay/ intellectual disability. Further genomic based testing may be considered if there remains a high suspicion of a monogenic disorder. Copy number variants that do not contain genes, are well established polymorphisms, or are assessed as being of unlikely clinical significance (based on "ACMG Technical Standards for the Interpretation and Reporting of Constitutional Copy Number Variants"), will not be reported. The classification is based on the current scientific evidence available at the time of reporting. Reporting of regions of homozygosity (>5Mb) is dependent on referral setting and clinical indication. CNVs that contain autosomal recessive genes will not be reported unless there is specific clinical relevance, high carrier population frequency or a history of consanguinity. Please contact the laboratory if there is a family history of a known recessive disorder or a clinically suspected recessive condition. This testing was performed on a standard SNP microarray platform which may not have sufficient probe coverage to detect clinically relevant CNVs related to this patient's specific clinical features. Please contact the laboratory if a higher resolution microarray may be required for a specific gene or genetic condition. This test does not exclude the possibility of tissue limited mosaicism and further testing of an alternative tissue may be considered if clinically indicated. Interpretation is based on the UCSC GRCh38/hg38 human reference sequence.

Validated: 10-Nov-2023 by Vida Petrovic

Enquiries: +61 1300 11 8247

END OF TEST REPORT

FINAL REPORT

PATHOLOGY REPORT

Victorian Clinical Genetics Services
Murdoch Children's Research Institute
Flemington Road, Parkville, VIC 3052 Australia
P +61 1300 118247 F +61 3 8341 6366
W vcgs.org.au ABN 51 007 032 760

LIMS Report #: 961285

Patient: EO1 #87.4 P11 Eo1 #87.4 P11

Dr. Katerina Vlahos
Murdoch Children's Research Institute
Level 5/Flemington Road
PARKVILLE VIC 3052

DOB: Unknown
Sex: Unknown
VCGS sample ID: 22C111969
Date collected: 14-Dec-2022
Date received: 14-Dec-2022
Date reported: 29-Dec-2022
Source: Cell Pellet
Ext. sample ID:
Ext. patient ID:

CC: Ips.gene

Cytogenetics Laboratory

**Clinical details**	Cell line
**Specimen source**	Cell Pellet

Molecular karyotype

**Array type**	Illumina Infinium GSA-24 v3.0
**Resolution**	0.50Mb
**Assembly**	hg19 / GRCh37 (Feb 2009)
**Molecular karyotype**	arr(X,1-22)x2
**Result**	NO ANEUPLOIDIES DETECTED

Interpretation

Female molecular karyotype. No aneuploidies were detected in this sample.

Molecular karyotyping is limited in its ability to detect low grade mosaicism and genomic copy number changes below the resolution stated. Balanced rearrangements and Robertsonian translocations will not be detected. This test does not exclude single gene disorders caused by sequence mutations or trinucleotide repeat expansions (such as fragile X syndrome, Huntington disease, some spinocerebellar ataxias, Friedreich ataxia and myotonic dystrophy). Testing for fragile X syndrome should be considered in individuals with developmental delay/ intellectual disability. Further genomic based testing may be considered if there remains a high suspicion of a monogenic disorder. Copy number variants that do not contain genes, are well established polymorphisms, or are assessed as being of unlikely clinical significance (based on "ACMG Technical Standards for the Interpretation and Reporting of Constitutional Copy Number Variants"), will not be reported. The classification is based on the current scientific evidence available at the time of reporting. Reporting of regions of homozygosity (>5Mb) is dependent on referral setting and clinical indication. CNVs that contain autosomal recessive genes will not be reported unless there is specific clinical relevance, high carrier population frequency or a history of consanguinity. Please contact the laboratory if there is a family history of a known recessive disorder or a clinically suspected recessive condition. This testing was performed on a standard SNP microarray platform which may not have sufficient probe coverage to detect clinically relevant CNVs related to this patient's specific clinical features. Please contact the laboratory if a higher resolution microarray may be required for a specific gene or genetic condition. This test does not exclude the possibility of tissue limited mosaicism and further testing of an alternative tissue may be considered if clinically indicated. Interpretation is based on the UCSC GRCh37/hg19 human reference sequence.

Validated: 29-Dec-2022 by David Francis

Enquiries: +61 1300 11 8247

END OF TEST REPORT

FINAL REPORT

PATHOLOGY REPORT

Victorian Clinical Genetics Services
Murdoch Children's Research Institute
Flemington Road, Parkville, VIC 3052 Australia
P +61 1300 118247 F +61 3 8341 6366
W vcgs.org.au ABN 51 007 032 760

LIMS Report #: 1061946

Patient: EOI #110.1 P11 Eoi #110.1 P11

Dr. Katerina Vlahos
Murdoch Children's Research Institute
Level 5/Flemington Road
PARKVILLE VIC 3052

DOB: Unknown
Sex: Unknown
VCGS sample ID: 23C112733
Date collected: 19-Oct-2023
Date received: 19-Oct-2023
Date reported: 10-Nov-2023
Source: Cell Pellet
Ext. sample ID:
Ext. patient ID:

CC: Ips.gene

Cytogenetics Laboratory

**Clinical details**	Cell line
**Specimen source**	Cell Pellet

Molecular karyotype

**Array type**	Illumina Infinium GSA-24 v3.0
**Resolution**	0.50Mb
**Reference genome**	GRCh38 / hg38 (Dec 2013)
**Molecular karyotype**	arr(X,1-22)x2
**Result**	NO ANEUPLOIDIES DETECTED

Interpretation

Female molecular karyotype. No aneuploidies were detected in this sample.

Molecular karyotyping is limited in its ability to detect low grade mosaicism and genomic copy number changes below the resolution stated. Balanced rearrangements and Robertsonian translocations will not be detected. This test does not exclude single gene disorders caused by sequence mutations or trinucleotide repeat expansions (such as fragile X syndrome, Huntington disease, some spinocerebellar ataxias, Friedreich ataxia and myotonic dystrophy). Testing for fragile X syndrome should be considered in individuals with developmental delay/ intellectual disability. Further genomic based testing may be considered if there remains a high suspicion of a monogenic disorder. Copy number variants that do not contain genes, are well established polymorphisms, or are assessed as being of unlikely clinical significance (based on "ACMG Technical Standards for the Interpretation and Reporting of Constitutional Copy Number Variants"), will not be reported. The classification is based on the current scientific evidence available at the time of reporting. Reporting of regions of homozygosity (>5Mb) is dependent on referral setting and clinical indication. CNVs that contain autosomal recessive genes will not be reported unless there is specific clinical relevance, high carrier population frequency or a history of consanguinity. Please contact the laboratory if there is a family history of a known recessive disorder or a clinically suspected recessive condition. This testing was performed on a standard SNP microarray platform which may not have sufficient probe coverage to detect clinically relevant CNVs related to this patient's specific clinical features. Please contact the laboratory if a higher resolution microarray may be required for a specific gene or genetic condition. This test does not exclude the possibility of tissue limited mosaicism and further testing of an alternative tissue may be considered if clinically indicated. Interpretation is based on the UCSC GRCh38/hg38 human reference sequence.

Validated: 10-Nov-2023 by Vida Petrovic

Enquiries: +61 1300 11 8247

END OF TEST REPORT

FINAL REPORT

PATHOLOGY REPORT

Victorian Clinical Genetics Services
Murdoch Children's Research Institute
Flemington Road, Parkville, VIC 3052 Australia
P +61 1300 118247 F +61 3 8341 6366
W vcgs.org.au ABN 51 007 032 760

LIMS Report #: 1010836

Patient: EOI#98.5 IPSC P11 Eoi#98.5 Ipsc P11

DOB: Unknown
Sex:
VCGS sample ID: 23C105301
Date collected: 10-May-2023
Date received: 10-May-2023
Date reported: 05-Jun-2023
Source: Cell Pellet
Ext. sample ID:
Ext. patient ID:

Dr. Katerina Vlahos
Murdoch Children's Research Institute
Level 5/Flemington Road
PARKVILLE VIC 3052

CC: Ips.gene

Cytogenetics Laboratory

**Clinical details**	Cell line
**Specimen source**	Cell Pellet

Molecular karyotype

**Array type**	Illumina Infinium GSA-24 v3.0
**Resolution**	0.50Mb
**Reference genome**	GRCh38 / hg38 (Dec 2013)
**Molecular karyotype**	arr(X,1-22)x2
**Result**	NO ANEUPLOIDIES DETECTED

Interpretation

Female molecular karyotype. No aneuploidies were detected in this sample.

Molecular karyotyping is limited in its ability to detect low grade mosaicism and genomic copy number changes below the resolution stated. Balanced rearrangements and Robertsonian translocations will not be detected. This test does not exclude single gene disorders caused by sequence mutations or trinucleotide repeat expansions (such as fragile X syndrome, Huntington disease, some spinocerebellar ataxias, Friedreich ataxia and myotonic dystrophy). Testing for fragile X syndrome should be considered in individuals with developmental delay/ intellectual disability. Further genomic based testing may be considered if there remains a high suspicion of a monogenic disorder. Copy number variants that do not contain genes, are well established polymorphisms, or are assessed as being of unlikely clinical significance (based on "ACMG Technical Standards for the Interpretation and Reporting of Constitutional Copy Number Variants"), will not be reported. The classification is based on the current scientific evidence available at the time of reporting. Reporting of regions of homozygosity (>5Mb) is dependent on referral setting and clinical indication. CNVs that contain autosomal recessive genes will not be reported unless there is specific clinical relevance, high carrier population frequency or a history of consanguinity. Please contact the laboratory if there is a family history of a known recessive disorder or a clinically suspected recessive condition. CNVs involving moderate to high risk cancer susceptibility genes may be reported. However, incidentally ascertained CNVs involving genes assessed as having low to moderate cancer risk will not generally be reported unless they are part of a larger CNV. This testing was performed on a standard SNP microarray platform which may not have sufficient probe coverage to detect clinically relevant CNVs related to this patient's specific clinical features. Please contact the laboratory if a higher resolution microarray may be required for a specific gene or genetic condition. This test does not exclude the possibility of tissue limited mosaicism and further testing of an alternative tissue may be considered if clinically indicated. Interpretation is based on the UCSC GRCh38/hg38 human reference sequence.

Validated: 05-Jun-2023 by David Francis

Enquiries: +61 1300 11 8247

PATHOLOGY REPORT

END OF TEST REPORT

FINAL REPORT

Victorian Clinical Genetics Services
Murdoch Children's Research Institute
Flemington Road, Parkville, VIC 3052 Australia
P +61 1300 118247 F +61 3 8341 6366
W vcgs.org.au ABN 51 007 032 760

LIMS Report #: 1018770

Patient: BD009.2 IPSC P11 Bd009.2 Ipsc P11

Dr. Katerina Vlahos
Murdoch Children's Research Institute
Level 5/Flemington Road
PARKVILLE VIC 3052

DOB: Unknown
Sex: Unknown
VCGS sample ID: 23C106576
Date collected: 06-Jun-2023
Date received: 06-Jun-2023
Date reported: 29-Jun-2023
Source: Cell Pellet
Ext. sample ID:
Ext. patient ID:

Cytogenetics Laboratory

**Clinical details**	Cell line
**Specimen source**	Cell Pellet

Molecular karyotype

**Array type**	Illumina Infinium GSA-24 v3.0
**Resolution**	0.50Mb
**Reference genome**	GRCh38 / hg38 (Dec 2013)
**Molecular karyotype**	arr(X,Y)x1,(1-22)x2
**Result**	NO ANEUPLOIDIES DETECTED

Interpretation

Male molecular karyotype. No aneuploidies were detected in this sample.

Molecular karyotyping is limited in its ability to detect low grade mosaicism and genomic copy number changes below the resolution stated. Balanced rearrangements and Robertsonian translocations will not be detected. This test does not exclude single gene disorders caused by sequence mutations or trinucleotide repeat expansions (such as fragile X syndrome, Huntington disease, some spinocerebellar ataxias, Friedreich ataxia and myotonic dystrophy). Testing for fragile X syndrome should be considered in individuals with developmental delay/ intellectual disability. Further genomic based testing may be considered if there remains a high suspicion of a monogenic disorder. Copy number variants that do not contain genes, are well established polymorphisms, or are assessed as being of unlikely clinical significance (based on “ACMG Technical Standards for the Interpretation and Reporting of Constitutional Copy Number Variants”), will not be reported. The classification is based on the current scientific evidence available at the time of reporting. Reporting of regions of homozygosity (>5Mb) is dependent on referral setting and clinical indication. CNVs that contain autosomal recessive genes will not be reported unless there is specific clinical relevance, high carrier population frequency or a history of consanguinity. Please contact the laboratory if there is a family history of a known recessive disorder or a clinically suspected recessive condition. CNVs involving moderate to high risk cancer susceptibility genes may be reported. However, incidentally ascertained CNVs involving genes assessed as having low to moderate cancer risk will not generally be reported unless they are part of a larger CNV. This testing was performed on a standard SNP microarray platform which may not have sufficient probe coverage to detect clinically relevant CNVs related to this patient’s specific clinical features. Please contact the laboratory if a higher resolution microarray may be required for a specific gene or genetic condition. This test does not exclude the possibility of tissue limited mosaicism and further testing of an alternative tissue may be considered if clinically indicated. Interpretation is based on the UCSC GRCh38/hg38 human reference sequence.

Validated: 29-Jun-2023 by David Francis

Enquiries: +61 1300 11 8247

PATHOLOGY REPORT

END OF TEST REPORT

FINAL REPORT

Victorian Clinical Genetics Services
Murdoch Children's Research Institute
Flemington Road, Parkville, VIC 3052 Australia
P +61 1300 118247 F +61 3 8341 6366
W vcgs.org.au ABN 51 007 032 760

VCGS leaders in genetic health

LIMS Report #: 850338

Patient: BD001 P11 Bd001 P11

DOB: Unknown
Sex: Unknown
VCGS sample ID: 21C111367
Date collected: 14-Dec-2021
Date received: 14-Dec-2021
Date reported: 05-Jan-2022
Source: Cell Pellet
Ext. sample ID:
Ext. patient ID:

Dr. Katerina Vlahos
Murdoch Childrens Research Institute
Level 5/Flemington Road
PARKVILLE VIC 3052

Cytogenetics Laboratory

**Clinical details**	Cell line
**Specimen source**	Cell Pellet

Molecular karyotype

**Array type**	Illumina Infinium GSA-24 v3.0
**Resolution**	0.50Mb
**Assembly**	hg19 / GRCh37 (Feb 2009)
**Molecular karyotype**	arr(X,1-22)x2
**Result**	NO ANEUPLOIDIES DETECTED

Interpretation

Female molecular karyotype. No aneuploidies were detected in this sample.

Molecular karyotyping is limited in its ability to detect low grade mosaicism and genomic copy number changes below the resolution stated. Balanced rearrangements and Robertsonian translocations will not be detected. This test does not exclude single gene disorders caused by sequence mutations or trinucleotide repeat expansions (such as fragile X syndrome, Huntington disease, some spinocerebellar ataxias, Friedreich ataxia and myotonic dystrophy). Testing for fragile X syndrome should be considered in individuals with developmental delay/ intellectual disability. Further genomic based testing may be considered if there remains a high suspicion of a monogenic disorder. Copy number variants that do not contain genes, are well established polymorphisms, or are assessed as being of unlikely clinical significance (based on "ACMG Technical Standards for the Interpretation and Reporting of Constitutional Copy Number Variants"), will not be reported. The classification is based on the current scientific evidence available at the time of reporting. Reporting of regions of homozygosity (>5Mb) is dependent on referral setting and clinical indication. CNVs that contain autosomal recessive genes will not be reported unless there is specific clinical relevance, high carrier population frequency or a history of consanguinity. Please contact the laboratory if there is a family history of a known recessive disorder or a clinically suspected recessive condition. This testing was performed on a standard SNP microarray platform which may not have sufficient probe coverage to detect clinically relevant CNVs related to this patient's specific clinical features. Please contact the laboratory if a higher resolution microarray may be required for a specific gene or genetic condition. This test does not exclude the possibility of tissue limited mosaicism and further testing of an alternative tissue may be considered if clinically indicated. Interpretation is based on the UCSC GRCh37/hg19 human reference sequence.

Validated: 05-Jan-2022 by David Francis

Enquiries: +61 1300 11 8247

PATHOLOGY REPORT

END OF TEST REPORT

FINAL REPORT

Victorian Clinical Genetics Services
Murdoch Children's Research Institute
Flemington Road, Parkville, VIC 3052 Australia
P +61 1300 118247 F +61 3 8341 6366
W vcgs.org.au ABN 51 007 032 760

LIMS Report #: 922190

Patient: BD004.9 IPSC P10 Bd004.9 Ipsc P10

Dr. Katerina Vlahos
Murdoch Children's Research Institute
Level 5/Flemington Road
PARKVILLE VIC 3052

DOB: Unknown
Sex: Unknown
VCGS sample ID: 22C107158
Date collected: 10-Aug-2022
Date received: 10-Aug-2022
Date reported: 23-Aug-2022
Source: Cell Pellet
Ext. sample ID:
Ext. patient ID:

CC: Ips.gene

Cytogenetics Laboratory

**Clinical details**	Cell line
**Specimen source**	Cell Pellet

Molecular karyotype

**Array type**	Illumina Infinium GSA-24 v3.0
**Resolution**	0.50Mb
**Assembly**	hg19 / GRCh37 (Feb 2009)
**Molecular karyotype**	arr(X,1-22)x2
**Result**	NO ANEUPLOIDIES DETECTED

Interpretation

Female molecular karyotype. No aneuploidies were detected in this sample.

Molecular karyotyping is limited in its ability to detect low grade mosaicism and genomic copy number changes below the resolution stated. Balanced rearrangements and Robertsonian translocations will not be detected. This test does not exclude single gene disorders caused by sequence mutations or trinucleotide repeat expansions (such as fragile X syndrome, Huntington disease, some spinocerebellar ataxias, Friedreich ataxia and myotonic dystrophy). Testing for fragile X syndrome should be considered in individuals with developmental delay/ intellectual disability. Further genomic based testing may be considered if there remains a high suspicion of a monogenic disorder. Copy number variants that do not contain genes, are well established polymorphisms, or are assessed as being of unlikely clinical significance (based on "ACMG Technical Standards for the Interpretation and Reporting of Constitutional Copy Number Variants"), will not be reported. The classification is based on the current scientific evidence available at the time of reporting. Reporting of regions of homozygosity (>5Mb) is dependent on referral setting and clinical indication. CNVs that contain autosomal recessive genes will not be reported unless there is specific clinical relevance, high carrier population frequency or a history of consanguinity. Please contact the laboratory if there is a family history of a known recessive disorder or a clinically suspected recessive condition. This testing was performed on a standard SNP microarray platform which may not have sufficient probe coverage to detect clinically relevant CNVs related to this patient's specific clinical features. Please contact the laboratory if a higher resolution microarray may be required for a specific gene or genetic condition. This test does not exclude the possibility of tissue limited mosaicism and further testing of an alternative tissue may be considered if clinically indicated. Interpretation is based on the UCSC GRCh37/hg19 human reference sequence.

Validated: 23-Aug-2022 by David Francis

Enquiries: +61 1300 11 8247

PATHOLOGY REPORT

END OF TEST REPORT

FINAL REPORT

Victorian Clinical Genetics Services
Murdoch Children's Research Institute
Flemington Road, Parkville, VIC 3052 Australia
P +61 1300 118247 F +61 3 8341 6366
W vcgs.org.au ABN 51 007 032 760

LIMS Report #: 850376

Patient: BD012.2 P11 Bd012.2 P11

Dr. Katerina Vlahos
Murdoch Childrens Research Institute
Level 5/Flemington Road
PARKVILLE VIC 3052

DOB: Unknown
Sex: Unknown
VCGS sample ID: 21C111373
Date collected: 14-Dec-2021
Date received: 14-Dec-2021
Date reported: 05-Jan-2022
Source: Cell Pellet
Ext. sample ID:
Ext. patient ID:

Cytogenetics Laboratory

**Clinical details**	Cell line
**Specimen source**	Cell Pellet

Molecular karyotype

**Array type**	Illumina Infinium GSA-24 v3.0
**Resolution**	0.50Mb
**Assembly**	hg19 / GRCh37 (Feb 2009)
**Molecular karyotype**	arr(X,Y)x1,(1-22)x2
**Result**	NO ANEUPLOIDIES DETECTED

Interpretation

Male molecular karyotype. No aneuploidies were detected in this sample.

Molecular karyotyping is limited in its ability to detect low grade mosaicism and genomic copy number changes below the resolution stated. Balanced rearrangements and Robertsonian translocations will not be detected. This test does not exclude single gene disorders caused by sequence mutations or trinucleotide repeat expansions (such as fragile X syndrome, Huntington disease, some spinocerebellar ataxias, Friedreich ataxia and myotonic dystrophy). Testing for fragile X syndrome should be considered in individuals with developmental delay/ intellectual disability. Further genomic based testing may be considered if there remains a high suspicion of a monogenic disorder. Copy number variants that do not contain genes, are well established polymorphisms, or are assessed as being of unlikely clinical significance (based on "ACMG Technical Standards for the Interpretation and Reporting of Constitutional Copy Number Variants"), will not be reported. The classification is based on the current scientific evidence available at the time of reporting. Reporting of regions of homozygosity (>5Mb) is dependent on referral setting and clinical indication. CNVs that contain autosomal recessive genes will not be reported unless there is specific clinical relevance, high carrier population frequency or a history of consanguinity. Please contact the laboratory if there is a family history of a known recessive disorder or a clinically suspected recessive condition. This testing was performed on a standard SNP microarray platform which may not have sufficient probe coverage to detect clinically relevant CNVs related to this patient's specific clinical features. Please contact the laboratory if a higher resolution microarray may be required for a specific gene or genetic condition. This test does not exclude the possibility of tissue limited mosaicism and further testing of an alternative tissue may be considered if clinically indicated. Interpretation is based on the UCSC GRCh37/hg19 human reference sequence.

Validated: 05-Jan-2022 by David Francis

Enquiries: +61 1300 11 8247

PATHOLOGY REPORT

END OF TEST REPORT

FINAL REPORT

Victorian Clinical Genetics Services
Murdoch Children's Research Institute
Flemington Road, Parkville, VIC 3052 Australia
P +61 1300 118247 F +61 3 8341 6366
W vcgs.org.au ABN 51 007 032 760

LIMS Report #: 1018767

Patient: BD005.5 IPSC P11 Bd005.5 Ipsc P11

DOB: Unknown
Sex: Unknown
VCGS sample ID: 23C106574
Date collected: 06-Jun-2023
Date received: 06-Jun-2023
Date reported: 29-Jun-2023
Source: Cell Pellet
Ext. sample ID:
Ext. patient ID:

Dr. Katerina Vlahos
Murdoch Children's Research Institute
Level 5/Flemington Road
PARKVILLE VIC 3052

CC: Ips.gene

PATHOLOGY REPORT

Cytogenetics Laboratory

**Clinical details**	Cell line
**Specimen source**	Cell Pellet

Molecular karyotype

**Array type**	Illumina Infinium GSA-24 v3.0
**Resolution**	0.50Mb
**Reference genome**	GRCh38 / hg38 (Dec 2013)
**Molecular karyotype**	arr(X,Y)x1,(1-22)x2
**Result**	NO ANEUPLOIDIES DETECTED

Interpretation

Male molecular karyotype. No aneuploidies were detected in this sample.

Molecular karyotyping is limited in its ability to detect low grade mosaicism and genomic copy number changes below the resolution stated. Balanced rearrangements and Robertsonian translocations will not be detected. This test does not exclude single gene disorders caused by sequence mutations or trinucleotide repeat expansions (such as fragile X syndrome, Huntington disease, some spinocerebellar ataxias, Friedreich ataxia and myotonic dystrophy). Testing for fragile X syndrome should be considered in individuals with developmental delay/ intellectual disability. Further genomic based testing may be considered if there remains a high suspicion of a monogenic disorder. Copy number variants that do not contain genes, are well established polymorphisms, or are assessed as being of unlikely clinical significance (based on "ACMG Technical Standards for the Interpretation and Reporting of Constitutional Copy Number Variants"), will not be reported. The classification is based on the current scientific evidence available at the time of reporting. Reporting of regions of homozygosity (>5Mb) is dependent on referral setting and clinical indication. CNVs that contain autosomal recessive genes will not be reported unless there is specific clinical relevance, high carrier population frequency or a history of consanguinity. Please contact the laboratory if there is a family history of a known recessive disorder or a clinically suspected recessive condition. CNVs involving moderate to high risk cancer susceptibility genes may be reported. However, incidentally ascertained CNVs involving genes assessed as having low to moderate cancer risk will not generally be reported unless they are part of a larger CNV. This testing was performed on a standard SNP microarray platform which may not have sufficient probe coverage to detect clinically relevant CNVs related to this patient's specific clinical features. Please contact the laboratory if a higher resolution microarray may be required for a specific gene or genetic condition. This test does not exclude the possibility of tissue limited mosaicism and further testing of an alternative tissue may be considered if clinically indicated. Interpretation is based on the UCSC GRCh38/hg38 human reference sequence.

Validated: 29-Jun-2023 by David Francis

Enquiries: +61 1300 11 8247

END OF TEST REPORT

FINAL REPORT

Victorian Clinical Genetics Services
Murdoch Children's Research Institute
Flemington Road, Parkville, VIC 3052 Australia
P +61 1300 118247 F +61 3 8341 6366
W vcgs.org.au ABN 51 007 032 760

LIMS Report #: 901661

Patient: BD002.3 P10 Bd002.3 P10

Dr. Katerina Vlahos
Murdoch Children's Research Institute
Level 5/Flemington Road
PARKVILLE VIC 3052

DOB: Unknown
Sex: Unknown
VCGS sample ID: 22C103779
Date collected: 05-May-2022
Date received: 05-May-2022
Date reported: 20-Jun-2022
Source: Cell Pellet
Ext. sample ID:
Ext. patient ID:

CC: Ips.gene

Cytogenetics Laboratory

**Clinical details**	Cell line
**Specimen source**	Cell Pellet

Molecular karyotype

**Array type**	Illumina Infinium GSA-24 v3.0
**Resolution**	0.50Mb
**Assembly**	hg19 / GRCh37 (Feb 2009)
**Molecular karyotype**	arr(X,1-22)x2
**Result**	NO ANEUPLOIDIES DETECTED

Interpretation

Female molecular karyotype. No aneuploidies were detected in this sample.

Molecular karyotyping is limited in its ability to detect low grade mosaicism and genomic copy number changes below the resolution stated. Balanced rearrangements and Robertsonian translocations will not be detected. This test does not exclude single gene disorders caused by sequence mutations or trinucleotide repeat expansions (such as fragile X syndrome, Huntington disease, some spinocerebellar ataxias, Friedreich ataxia and myotonic dystrophy). Testing for fragile X syndrome should be considered in individuals with developmental delay/ intellectual disability. Further genomic based testing may be considered if there remains a high suspicion of a monogenic disorder. Copy number variants that do not contain genes, are well established polymorphisms, or are assessed as being of unlikely clinical significance (based on "ACMG Technical Standards for the Interpretation and Reporting of Constitutional Copy Number Variants"), will not be reported. The classification is based on the current scientific evidence available at the time of reporting. Reporting of regions of homozygosity (>5Mb) is dependent on referral setting and clinical indication. CNVs that contain autosomal recessive genes will not be reported unless there is specific clinical relevance, high carrier population frequency or a history of consanguinity. Please contact the laboratory if there is a family history of a known recessive disorder or a clinically suspected recessive condition. This testing was performed on a standard SNP microarray platform which may not have sufficient probe coverage to detect clinically relevant CNVs related to this patient's specific clinical features. Please contact the laboratory if a higher resolution microarray may be required for a specific gene or genetic condition. This test does not exclude the possibility of tissue limited mosaicism and further testing of an alternative tissue may be considered if clinically indicated. Interpretation is based on the UCSC GRCh37/hg19 human reference sequence.

Validated: 20-Jun-2022 by David Francis

Enquiries: +61 1300 11 8247

END OF TEST REPORT

FINAL REPORT

PATHOLOGY REPORT

Victorian Clinical Genetics Services
Murdoch Children's Research Institute
Flemington Road, Parkville, VIC 3052 Australia
P +61 1300 118247 F +61 3 8341 6366
W vcgs.org.au ABN 51 007 032 760

LIMS Report #: 850370

Patient: BD010.5 P10 Bd010.5 P10

Dr. Katerina Vlahos
Murdoch Childrens Research Institute
Level 5/Flemington Road
PARKVILLE VIC 3052

DOB: Unknown
Sex: Unknown
VCGS sample ID: 21C111372
Date collected: 14-Dec-2021
Date received: 14-Dec-2021
Date reported: 05-Jan-2022
Source: Cell Pellet
Ext. sample ID:
Ext. patient ID:

Cytogenetics Laboratory

PATHOLOGY REPORT

**Clinical details**	Cell line
**Specimen source**	Cell Pellet

Molecular karyotype

**Array type**	Illumina Infinium GSA-24 v3.0
**Resolution**	0.50Mb
**Assembly**	hg19 / GRCh37 (Feb 2009)
**Molecular karyotype**	arr(X,1-22)x2
**Result**	NO ANEUPLOIDIES DETECTED

Interpretation

Female molecular karyotype. No aneuploidies were detected in this sample.

Molecular karyotyping is limited in its ability to detect low grade mosaicism and genomic copy number changes below the resolution stated. Balanced rearrangements and Robertsonian translocations will not be detected. This test does not exclude single gene disorders caused by sequence mutations or trinucleotide repeat expansions (such as fragile X syndrome, Huntington disease, some spinocerebellar ataxias, Friedreich ataxia and myotonic dystrophy). Testing for fragile X syndrome should be considered in individuals with developmental delay/ intellectual disability. Further genomic based testing may be considered if there remains a high suspicion of a monogenic disorder. Copy number variants that do not contain genes, are well established polymorphisms, or are assessed as being of unlikely clinical significance (based on "ACMG Technical Standards for the Interpretation and Reporting of Constitutional Copy Number Variants"), will not be reported. The classification is based on the current scientific evidence available at the time of reporting. Reporting of regions of homozygosity (>5Mb) is dependent on referral setting and clinical indication. CNVs that contain autosomal recessive genes will not be reported unless there is specific clinical relevance, high carrier population frequency or a history of consanguinity. Please contact the laboratory if there is a family history of a known recessive disorder or a clinically suspected recessive condition. This testing was performed on a standard SNP microarray platform which may not have sufficient probe coverage to detect clinically relevant CNVs related to this patient's specific clinical features. Please contact the laboratory if a higher resolution microarray may be required for a specific gene or genetic condition. This test does not exclude the possibility of tissue limited mosaicism and further testing of an alternative tissue may be considered if clinically indicated. Interpretation is based on the UCSC GRCh37/hg19 human reference sequence.

Validated: 05-Jan-2022 by David Francis

Enquiries: +61 1300 11 8247

END OF TEST REPORT

FINAL REPORT

Accredited for compliance with NPAAC Standards and ISO 15189
Accreditation number: 3171

Victorian Clinical Genetics Services
Murdoch Children's Research Institute
Flemington Road, Parkville, VIC 3052 Australia
P +61 1300 118247 F +61 3 8341 6366
W vcgs.org.au ABN 51 007 032 760

LIMS Report #: 850369

Patient: BD008.2 P10 Bd008.2 P10

Dr. Katerina Vlahos
Murdoch Childrens Research Institute
Level 5/Flemington Road
PARKVILLE VIC 3052

DOB: Unknown
Sex: Unknown
VCGS sample ID: 21C111371
Date collected: 14-Dec-2021
Date received: 14-Dec-2021
Date reported: 05-Jan-2022
Source: Cell Pellet
Ext. sample ID:
Ext. patient ID:

Cytogenetics Laboratory

**Clinical details**	Cell line
**Specimen source**	Cell Pellet

Molecular karyotype

**Array type**	Illumina Infinium GSA-24 v3.0
**Resolution**	0.50Mb
**Assembly**	hg19 / GRCh37 (Feb 2009)
**Molecular karyotype**	arr(X,1-22)x2
**Result**	NO ANEUPLOIDIES DETECTED

Interpretation

Female molecular karyotype. No aneuploidies were detected in this sample.

Molecular karyotyping is limited in its ability to detect low grade mosaicism and genomic copy number changes below the resolution stated. Balanced rearrangements and Robertsonian translocations will not be detected. This test does not exclude single gene disorders caused by sequence mutations or trinucleotide repeat expansions (such as fragile X syndrome, Huntington disease, some spinocerebellar ataxias, Friedreich ataxia and myotonic dystrophy). Testing for fragile X syndrome should be considered in individuals with developmental delay/ intellectual disability. Further genomic based testing may be considered if there remains a high suspicion of a monogenic disorder. Copy number variants that do not contain genes, are well established polymorphisms, or are assessed as being of unlikely clinical significance (based on "ACMG Technical Standards for the Interpretation and Reporting of Constitutional Copy Number Variants"), will not be reported. The classification is based on the current scientific evidence available at the time of reporting. Reporting of regions of homozygosity (>5Mb) is dependent on referral setting and clinical indication. CNVs that contain autosomal recessive genes will not be reported unless there is specific clinical relevance, high carrier population frequency or a history of consanguinity. Please contact the laboratory if there is a family history of a known recessive disorder or a clinically suspected recessive condition. This testing was performed on a standard SNP microarray platform which may not have sufficient probe coverage to detect clinically relevant CNVs related to this patient's specific clinical features. Please contact the laboratory if a higher resolution microarray may be required for a specific gene or genetic condition. This test does not exclude the possibility of tissue limited mosaicism and further testing of an alternative tissue may be considered if clinically indicated. Interpretation is based on the UCSC GRCh37/hg19 human reference sequence.

Validated: 05-Jan-2022 by David Francis

Enquiries: +61 1300 11 8247

PATHOLOGY REPORT

END OF TEST REPORT

FINAL REPORT

Victorian Clinical Genetics Services
Murdoch Children's Research Institute
Flemington Road, Parkville, VIC 3052 Australia
P +61 1300 118247 F +61 3 8341 6366
W vcgs.org.au ABN 51 007 032 760

VCGS leaders in genetic health

LIMS Report #: 901645

Patient: BD003.1 P12 Bd003.1 P12

DOB: Unknown
Sex: Unknown
VCGS sample ID: 22C103775
Date collected: 05-May-2022
Date received: 05-May-2022
Date reported: 20-Jun-2022
Source: Cell Pellet
Ext. sample ID:
Ext. patient ID:

Dr. Katerina Vlahos
Murdoch Children's Research Institute
Level 5/Flemington Road
PARKVILLE VIC 3052

CC: Ips.gene

Cytogenetics Laboratory

**Clinical details**	Cell line
**Specimen source**	Cell Pellet

Molecular karyotype

**Array type**	Illumina Infinium GSA-24 v3.0
**Resolution**	0.50Mb
**Assembly**	hg19 / GRCh37 (Feb 2009)
**Molecular karyotype**	arr(X,1-22)x2
**Result**	NO ANEUPLOIDIES DETECTED

Interpretation

Female molecular karyotype. No aneuploidies were detected in this sample.

Molecular karyotyping is limited in its ability to detect low grade mosaicism and genomic copy number changes below the resolution stated. Balanced rearrangements and Robertsonian translocations will not be detected. This test does not exclude single gene disorders caused by sequence mutations or trinucleotide repeat expansions (such as fragile X syndrome, Huntington disease, some spinocerebellar ataxias, Friedreich ataxia and myotonic dystrophy). Testing for fragile X syndrome should be considered in individuals with developmental delay/ intellectual disability. Further genomic based testing may be considered if there remains a high suspicion of a monogenic disorder. Copy number variants that do not contain genes, are well established polymorphisms, or are assessed as being of unlikely clinical significance (based on "ACMG Technical Standards for the Interpretation and Reporting of Constitutional Copy Number Variants"), will not be reported. The classification is based on the current scientific evidence available at the time of reporting. Reporting of regions of homozygosity (>5Mb) is dependent on referral setting and clinical indication. CNVs that contain autosomal recessive genes will not be reported unless there is specific clinical relevance, high carrier population frequency or a history of consanguinity. Please contact the laboratory if there is a family history of a known recessive disorder or a clinically suspected recessive condition. This testing was performed on a standard SNP microarray platform which may not have sufficient probe coverage to detect clinically relevant CNVs related to this patient's specific clinical features. Please contact the laboratory if a higher resolution microarray may be required for a specific gene or genetic condition. This test does not exclude the possibility of tissue limited mosaicism and further testing of an alternative tissue may be considered if clinically indicated. Interpretation is based on the UCSC GRCh37/hg19 human reference sequence.

Validated: 20-Jun-2022 by David Francis

Enquiries: +61 1300 11 8247

END OF TEST REPORT

FINAL REPORT

PATHOLOGY REPORT

Victorian Clinical Genetics Services
Murdoch Children's Research Institute
Flemington Road, Parkville, VIC 3052 Australia
P +61 1300 118247 F +61 3 8341 6366
W vcgs.org.au ABN 51 007 032 760

LIMS Report #: 850368

Patient: BD007.8 P11 Bd007.8 P11

Dr. Katerina Vlahos
Murdoch Childrens Research Institute
Level 5/Flemington Road
PARKVILLE VIC 3052

DOB: Unknown
Sex: Unknown
VCGS sample ID: 21C111370
Date collected: 14-Dec-2021
Date received: 14-Dec-2021
Date reported: 05-Jan-2022
Source: Cell Pellet
Ext. sample ID:
Ext. patient ID:

Cytogenetics Laboratory

**Clinical details**	Cell line
**Specimen source**	Cell Pellet

Molecular karyotype

**Array type**	Illumina Infinium GSA-24 v3.0
**Resolution**	0.50Mb
**Assembly**	hg19 / GRCh37 (Feb 2009)
**Molecular karyotype**	arr(X,1-22)x2
**Result**	NO ANEUPLOIDIES DETECTED

Interpretation

Female molecular karyotype. No aneuploidies were detected in this sample.

Molecular karyotyping is limited in its ability to detect low grade mosaicism and genomic copy number changes below the resolution stated. Balanced rearrangements and Robertsonian translocations will not be detected. This test does not exclude single gene disorders caused by sequence mutations or trinucleotide repeat expansions (such as fragile X syndrome, Huntington disease, some spinocerebellar ataxias, Friedreich ataxia and myotonic dystrophy). Testing for fragile X syndrome should be considered in individuals with developmental delay/ intellectual disability. Further genomic based testing may be considered if there remains a high suspicion of a monogenic disorder. Copy number variants that do not contain genes, are well established polymorphisms, or are assessed as being of unlikely clinical significance (based on "ACMG Technical Standards for the Interpretation and Reporting of Constitutional Copy Number Variants"), will not be reported. The classification is based on the current scientific evidence available at the time of reporting. Reporting of regions of homozygosity (>5Mb) is dependent on referral setting and clinical indication. CNVs that contain autosomal recessive genes will not be reported unless there is specific clinical relevance, high carrier population frequency or a history of consanguinity. Please contact the laboratory if there is a family history of a known recessive disorder or a clinically suspected recessive condition. This testing was performed on a standard SNP microarray platform which may not have sufficient probe coverage to detect clinically relevant CNVs related to this patient's specific clinical features. Please contact the laboratory if a higher resolution microarray may be required for a specific gene or genetic condition. This test does not exclude the possibility of tissue limited mosaicism and further testing of an alternative tissue may be considered if clinically indicated. Interpretation is based on the UCSC GRCh37/hg19 human reference sequence.

Validated: 05-Jan-2022 by David Francis

Enquiries: +61 1300 11 8247

PATHOLOGY REPORT

END OF TEST REPORT

FINAL REPORT

Accredited for compliance with NPAAC Standards and ISO 15189
Accreditation number: 3171

Victorian Clinical Genetics Services
Murdoch Children's Research Institute
Flemington Road, Parkville, VIC 3052 Australia
P +61 1300 118247 F +61 3 8341 6366
W vcgs.org.au ABN 51 007 032 760

LIMS Report #: 1018769

Patient: BD017.7 IPSC P11 Bd017.7 Ipsc P11

DOB: Unknown
Sex: Unknown
VCGS sample ID: 23C106575
Date collected: 06-Jun-2023
Date received: 06-Jun-2023
Date reported: 29-Jun-2023
Source: Cell Pellet
Ext. sample ID:
Ext. patient ID:

Dr. Katerina Vlahos
Murdoch Children's Research Institute
Level 5/Flemington Road
PARKVILLE VIC 3052

CC: Ips.gene

Cytogenetics Laboratory

**Clinical details**	Cell line
**Specimen source**	Cell Pellet

Molecular karyotype

**Array type**	Illumina Infinium GSA-24 v3.0
**Resolution**	0.50Mb
**Reference genome**	GRCh38 / hg38 (Dec 2013)
**Molecular karyotype**	arr(X,1-22)x2
**Result**	NO ANEUPLOIDIES DETECTED

Interpretation

Female molecular karyotype. No aneuploidies were detected in this sample.

Molecular karyotyping is limited in its ability to detect low grade mosaicism and genomic copy number changes below the resolution stated. Balanced rearrangements and Robertsonian translocations will not be detected. This test does not exclude single gene disorders caused by sequence mutations or trinucleotide repeat expansions (such as fragile X syndrome, Huntington disease, some spinocerebellar ataxias, Friedreich ataxia and myotonic dystrophy). Testing for fragile X syndrome should be considered in individuals with developmental delay/ intellectual disability. Further genomic based testing may be considered if there remains a high suspicion of a monogenic disorder. Copy number variants that do not contain genes, are well established polymorphisms, or are assessed as being of unlikely clinical significance (based on "ACMG Technical Standards for the Interpretation and Reporting of Constitutional Copy Number Variants"), will not be reported. The classification is based on the current scientific evidence available at the time of reporting. Reporting of regions of homozygosity (>5Mb) is dependent on referral setting and clinical indication. CNVs that contain autosomal recessive genes will not be reported unless there is specific clinical relevance, high carrier population frequency or a history of consanguinity. Please contact the laboratory if there is a family history of a known recessive disorder or a clinically suspected recessive condition. CNVs involving moderate to high risk cancer susceptibility genes may be reported. However, incidentally ascertained CNVs involving genes assessed as having low to moderate cancer risk will not generally be reported unless they are part of a larger CNV. This testing was performed on a standard SNP microarray platform which may not have sufficient probe coverage to detect clinically relevant CNVs related to this patient's specific clinical features. Please contact the laboratory if a higher resolution microarray may be required for a specific gene or genetic condition. This test does not exclude the possibility of tissue limited mosaicism and further testing of an alternative tissue may be considered if clinically indicated. Interpretation is based on the UCSC GRCh38/hg38 human reference sequence.

Validated: 29-Jun-2023 by David Francis

Enquiries: +61 1300 11 8247

END OF TEST REPORT

FINAL REPORT

PATHOLOGY REPORT

Victorian Clinical Genetics Services
Murdoch Children's Research Institute
Flemington Road, Parkville, VIC 3052 Australia
P +61 1300 118247 F +61 3 8341 6366
W vcgs.org.au ABN 51 007 032 760

VCGS
leaders in genetic health

LIMS Report #: 1018766

Patient: BD013.3 IPSC P11 Bd013.3 Ipsc P11

DOB: Unknown
Sex: Unknown
VCGS sample ID: 23C106573
Date collected: 06-Jun-2023
Date received: 06-Jun-2023
Date reported: 29-Jun-2023
Source: Cell Pellet
Ext. sample ID:
Ext. patient ID:

Dr. Katerina Vlahos
Murdoch Children's Research Institute
Level 5/Flemington Road
PARKVILLE VIC 3052

CC: Ips.gene

PATHOLOGY REPORT

Cytogenetics Laboratory

**Clinical details**	Cell line
**Specimen source**	Cell Pellet

Molecular karyotype

**Array type**	Illumina Infinium GSA-24 v3.0
**Resolution**	0.50Mb
**Reference genome**	GRCh38 / hg38 (Dec 2013)
**Molecular karyotype**	arr(X,1-22)x2
**Result**	NO ANEUPLOIDIES DETECTED

Interpretation

Female molecular karyotype. No aneuploidies were detected in this sample.

Molecular karyotyping is limited in its ability to detect low grade mosaicism and genomic copy number changes below the resolution stated. Balanced rearrangements and Robertsonian translocations will not be detected. This test does not exclude single gene disorders caused by sequence mutations or trinucleotide repeat expansions (such as fragile X syndrome, Huntington disease, some spinocerebellar ataxias, Friedreich ataxia and myotonic dystrophy). Testing for fragile X syndrome should be considered in individuals with developmental delay/ intellectual disability. Further genomic based testing may be considered if there remains a high suspicion of a monogenic disorder. Copy number variants that do not contain genes, are well established polymorphisms, or are assessed as being of unlikely clinical significance (based on "ACMG Technical Standards for the Interpretation and Reporting of Constitutional Copy Number Variants"), will not be reported. The classification is based on the current scientific evidence available at the time of reporting. Reporting of regions of homozygosity (>5Mb) is dependent on referral setting and clinical indication. CNVs that contain autosomal recessive genes will not be reported unless there is specific clinical relevance, high carrier population frequency or a history of consanguinity. Please contact the laboratory if there is a family history of a known recessive disorder or a clinically suspected recessive condition. CNVs involving moderate to high risk cancer susceptibility genes may be reported. However, incidentally ascertained CNVs involving genes assessed as having low to moderate cancer risk will not generally be reported unless they are part of a larger CNV. This testing was performed on a standard SNP microarray platform which may not have sufficient probe coverage to detect clinically relevant CNVs related to this patient's specific clinical features. Please contact the laboratory if a higher resolution microarray may be required for a specific gene or genetic condition. This test does not exclude the possibility of tissue limited mosaicism and further testing of an alternative tissue may be considered if clinically indicated. Interpretation is based on the UCSC GRCh38/hg38 human reference sequence.

Validated: 29-Jun-2023 by David Francis

Enquiries: +61 1300 11 8247

END OF TEST REPORT

FINAL REPORT

RCPA The Royal College of Pathologists of Australasia
NATA
Accredited for compliance with NPAAC Standards and ISO 15189
Accreditation number: 3171
